# Functional analysis of *HvSNAC1* in stomatal dynamics and drought adaptation

**DOI:** 10.1007/s13353-025-00956-6

**Published:** 2025-03-18

**Authors:** Marzena Kurowska, Agnieszka Janiak, Krzysztof Sitko, Izabela Potocka, Monika Gajecka, Ewa Sybilska, Tomasz Płociniczak, Sabina Lip, Magdalena Rynkiewicz, Klaudia Wiecha, Małgorzata Nawrot, Agata Daszkowska-Golec, Iwona Szarejko

**Affiliations:** Institute of Biology, Biotechnology and Environmental Protection, Faculty of Natural Sciences, University of Silesia in Katowice, Katowice, Poland

**Keywords:** Stomata density and reopening, TILLING, Barley, Drought, RNA-seq, HvSNAC1

## Abstract

Drought stress can damage crop growth and lead to a decline in yield, thereby affecting food security, especially in regions vulnerable to climate change. SNAC1 (stress-responsive NAC1), the NAC transcription factor family member, plays a crucial role in stomatal movement regulation. Effective regulation of stomatal movement is essential for protecting plants from water loss during adverse conditions. Our hypothesis revolves around altering HvSNAC1 activity by introducing a point mutation in its encoding gene, thereby influencing stomatal dynamics in barley. Two TILLING mutants, each harboring missense mutations in the NAC domain, exhibited higher stomatal density after drought stress compared to the parent cultivar ‘Sebastian’. These mutants also demonstrated distinct patterns of ABA-induced stomatal movement compared to the wild-type (WT). To delve deeper, we conducted a comprehensive analysis of the transcriptomes of these mutants and the parent cultivar ‘Sebastian’ under both optimal watering conditions and 10 days of drought stress treatment. We identified differentially expressed genes (DEGs) between the mutants and WT plants under control and drought conditions. Furthermore, we pinpointed DEGs specifically expressed in both mutants under drought conditions. Our experiments revealed that the *cis*-regulatory motif CACG, previously identified in Arabidopsis and rice, is recognized by HvSNAC1 in vitro. Enrichment analysis led to the identification of the cell wall organization category and potential target genes, such as *HvEXPA8* (*expansin 8*), *HvXTH* (*xyloglucan endotransglucosylase/hydrolase*), and *HvPAE9* (*pectin acetylesterase 9*), suggesting their regulation by HvSNAC1. These findings suggest that HvSNAC1 may play a role in regulating genes associated with stomatal density, size and reopening.

**Supplementary Information:**

The online version contains supplementary material available at 10.1007/s13353-025-00956-6.

## Introduction

Drought stress affects the yields of many plant species (Daryanto et al. 2016; Yang et al. 2020; Kim and Lee 2023). There are some predictions that the loss may be even higher because of climate change (Zhao et al. 2017). In the worst scenario, the global mean temperature might increase even by 5.7 °C in the 21st (Intergovernmental Panel on Climate Change 2022). There are some critical initiatives undertaken by plant scientists like Plants for climate ACTion! to tackle the climate crisis. They focus on finding immediate, mid-term and long-term solutions to counteract the consequences of climate change (Hirt et al. 2023). In plants, abiotic stress involves changes at the transcriptome, cellular and physiological levels (Atkinson and Urwin 2012; Osakabe et al. 2014). Plant responses are very complicated, and many mechanisms are simultaneously initiated to restore cellular homeostasis and promote survival (Golldack et al. 2014). It should be underlined that the molecular basis of drought response and the interactions between genes and proteins involved in these processes are not fully understood. Mutants generated by TILLING (targeting induced local lesion IN genome) method can help to understand many processes underlying the mechanism of drought response. Using this strategy, researchers can obtain a series of alleles in targeted genes with altered functions of encoded proteins in a short period (Kurowska et al. 2011a, b; Szurman-Zubrzycka et al. 2023).

Plant growth during water stress induces the expression of numerous transcription factors (TFs) that upregulate or downregulate a series of downstream genes. This cellular transcriptional network controls and modulates the stress-adaptive pathways. TFs are good candidate genes for the TILLING strategy because a single change in this complicated network could have an enormous impact on plant response to environmental stresses. One of the most essential plant transcription factor families is NAC. Proteins belonging to this family contain a highly conserved N-terminal dimerization and DNA-binding domain called the NAC domain, which is usually divided into five subdomains (A–E). This domain was initially characterized in petunia NAM and Arabidopsis ATAF1, ATAF2 and CUC2 (NAC) proteins (Ooka et al. 2003). The NAC domain is essential in recognizing and binding specifically to the NACRS (NAC recognized sequence) *cis*-acting elements to activate the corresponding gene expression. Several studies on the DNA binding activity of NAC domain proteins have shown significant variation in recognizing DNA sequence motifs in targeted genes. They mostly contain a CGT core sequence with varying numbers of surrounding consensus nucleotides. This has been reported for Arabidopsis (Olsen et al. 2005, Balazadeh et al. 2011; Welner et al. 2012; Wu et al. 2012), barley (Christiansen and Gregersen 2014) and wheat (Xue et al. 2006). In contrast, in Arabidopsis, the NACRS sequence was determined to be CACGCATCT (Tran et al. 2004). In rice, the CACG motif was found to be bound by SNAC1 in a few studies using a yeast one-hybrid assay (Hu et al. 2006; You et al. 2013; You et al. 2014a, b; Li et al. 2019). The newest research by Li et al. (2019), who employed the ChIP-Seq method, showed that SNAC1 preferably bound CACGT and CACGTA under normal growth conditions and to ACGTGG under drought conditions. Another characteristic feature of NAC proteins is the variable C-terminal transcriptional activation region (TAR), which confers activation or repression activity (Ooka et al. 2003). In some NAC proteins, these N- or C-terminal domains may modulate protein–protein interactions (Tran et al. 2010). In addition, transmembrane (TM) motifs for anchoring the protein to the plasma membrane are located in the C-terminal regions of some NACs proteins (Tran et al. 2010). NAC-type TFs have established enormously diverse roles in various plant developmental and morphogenic processes, including plant senescence control, secondary cell wall formation, cell cycle control and mixed biotic and abiotic stress responses (Ooka et al. 2003; Kim et al. 2006; Zhong et al. 2010; Nakashima et al. 2012; Puranik et al. 2013; Christiansen and Gregersen 2014; McGrann et al. 2015).

The gene *SNAC1 (stress-responsive NAC 1*) gene, a member of the NAC TF family that was identified for the first time in rice (Hu et al 2006), was the object of high interest after showing that its overexpression led to improved drought and salt tolerance in many plant species (reviewed in Kurowska and Daszkowska-Golec 2023). Its homologs have been identified in *Zea mays* (Liu et al. 2009), *Hordeum vulgare* (Kurowska et al. 2011b; Al Abdallat et al. 2014), *Sorghum bicolor* (Lu et al. 2012), *Eleusine coracana* (Ramegowda et al. 2012) and *Populus euphratica* (Liang et al. 2021). Many recent studies have demonstrated that transgenic plants overexpressing *SNAC1* or its homologs show improved drought and salt tolerance in *Arabidopsis thaliana* (Lu et al. 2013), *Triticum aestivum* (Saad et al. 2013), *Hordeum vulgare* (Al Abdallat et al. 2014), *Gossypim hirsutum* (Liu et al. 2014a, b), *Musa* sp. (Negi et al. 2021), *Nicotiana tabacum* (Ramegowda et al. 2012) and *Avena sativa* (Liang et al. 2021). *SNAC1* can be induced by drought, specifically in the guard cells in rice. Its expression can also be induced by salt, abscisic acid (ABA) and cold. In turn, *SNAC1-*overexpressing transgenic rice plants showed significantly improved drought resistance under field conditions and strong tolerance to salt stress (Hu et al. 2006). In detail, this response has been connected with delayed leaf rolling, higher number of closed stomata pores and lower transpiration rate resulting in slower rate of water loss, combined with the maintenance of photosynthesis rate. This resulted in significantly higher spikelet fertility under stress conditions (Hu et al. 2006). In rice, a comparative transcriptome analysis using microarrays between *SNAC1*-overexpressing plants and wild-type (WT) in control conditions showed that 80 genes were upregulated in *SNAC1*-overexpressing plants. Forty of these genes are related to drought stress protection mechanisms, such as specific signal transduction pathways, osmolyte production, detoxification and maintenance of redox homeostasis, protection of essential macromolecules from degradation and stomatal closure (Hu et al. 2006). Closing stomata faster to save water is one of the most intriguing results of *SNAC1*-overexpression in rice. These phenomena play a pivotal role in mitigating the impacts of abiotic stressors (Matkowski and Daszkowska-Golec 2023). Later research performed by Li et al. (2019) using global transcriptome analysis (RNA-Seq) and chromatin immunoprecipitation sequencing (ChIP-Seq) increased the knowledge of SNAC1 target genes at a genome-wide scale. Among the genes that have been identified by microarray or RNA-seq approaches, a few have been further confirmed by in vitro studies where SNAC1 was bound to its promoter sequence, e.g., *ERD1* (*early responsive to drought 1*) (Hu et al. 2006), *OsSRO1c* (*similar to RCD 1*) (You et al. 2013), *OsPP18* (*protein phosphatase 18*) (You et al. 2014a, b), *OsbZIP23* (Li et al. 2019), *OsNRT2.1* (*nitrate transporter 2.1*) (Qi et al. 2023). On this basis, the most critical processes that SNAC1 regulates through targeted genes are detoxification and redox homeostasis, stomatal closure, dephosphorylation, ABA sensitivity and nitrate uptake. Some mutants or overexpression lines were generated in genes targeted by SNAC1. The *erd1* mutant in Arabidopsis had defects in cell wall biosynthesis, which are associated with reduced shoot development and cell wall extensibility (Hsieh et al. 2023). The *ossro1c* mutant showed enhanced sensitivity to drought. Overexpression of *OsSRO1c* increased stomatal closure and reduced water loss by regulating hydrogen peroxide homeostasis (You et al. 2013). TaSRO1 in wheat has been shown to enhance seedling growth and abiotic stress resistance by modulating redox homeostasis, maintaining genomic integrity and fine-tuning the level of mitochondrial retrograde signaling (Liu et al. 2014a, b; Wang et al. 2022). The Arabidopsis *bzip19 bzip23* double mutant is hypersensitive to zinc deficiency (Assunção et al. 2010).

There is no available information on how the mutant in the *SNAC1* gene created through mutagenesis, including TILLING methods, responds to drought stress. However, Li et al. (2019) generated an *SNAC1*-knockout mutant and showed abnormal morphology and defects in spikelet fertility. Qi et al. (2023) showed decreased N uptake and a lower nitrogen use index (NUI) of the CRISPR/Cas9 mutant in the *SNAC1* gene.

Here, we used two mutants in the barley *HvSNAC1* gene that carried missense mutations in the NAC domain. The mutants were generated through the TILLING strategy to increase our understanding of the molecular mechanism of HvSNAC1 action in response to drought. We used the RNA-seq technique to identify a set of genes differentially expressed under drought compared to control conditions in the mutants and their parent cultivar ‘Sebastian’. The group of differentially expressed genes that were specifically downregulated in the mutants and not in the parent cultivar was more extensively analysed, as we assumed that mutations in the gene cause impairment of its action that may result in downregulation genes potentially regulated by HvSNAC1. The function of these genes is associated with the density and size of stomata as well as their reopening. Thus, HvSNAC1 may be involved in regulating of these features in barley.

## Materials and methods

### Plant material

The study used *Hor*TILLUS population (*Hordeum vulgare* – TILLING – University of Silesia) developed for the spring barley cultivar ‘Sebastian’ (Szurman-Zubrzycka et al. 2018). The 6021 M_2_ plants obtained after double treatment with sodium azide (NaN_3_) and N-methyl-N-nitrosourea (MNU) with 6 h of inter-incubation germination period (iig) were used for mutation screening.

### TILLING method

For the TILLING procedure, primers were designed to amplify a fragment of *HvSNAC1* gene that encompass 1277 bp of its sequence (F5′-CTCCTCTCACTCCCCAACAA-3′ and R5′-GTCATCCATTCCGCTTCTGT-3′). IRDye700 and IRDye800 labeled primers were used. The TILLING protocol has been described previously in detail (Szurman-Zubrzycka et al. 2018). Briefly, the PCR amplification was conducted in a 20-µl reaction volume composed of 14.4 µl of ddH_2_O, 1 × PCR buffer, 0.5 mM of dNTPs, 4 pmol of forward primer, 4 pmol of reverse primer, 0.5 U of color Taq polymerase and 150 ng of genomic DNA. Thermocycling conditions included an initial denaturation at 94 °C for 5 min, followed by 40 cycles of denaturation at 94 °C for 30 s, annealing at 58 °C for 45 s, extension at 72 °C for 1 min and 30 s and a final extension at 72 °C for 5 min. After amplification, heteroduplex formation was initiated with an initial denaturation at 95 °C for 3 min, followed by 70 cycles of slow renaturation starting from 70 °C for 20 s with a decrease of 0.1 °C per cycle. After heteroduplex formation, 10 µl of samples were treated with 20 µl of 0.1 × celery juice extract (CJE) containing Cel I enzyme. The enzymatic cleavage was performed at 45 °C for 15 min. The next step of the TILLING procedure was the purification of products by ethanol precipitation, which were later dissolve in 3 µl of formamide-containing buffer dedicated for polyacrylamide electrophoresis. Electrophoresis was performed on a LICOR 4300 or 4200 (LI-COR, Lincoln, NE, USA), and gel images were analysed using e-Seq software.

The mutation density for the *HvSNAC1* gene was estimated by dividing the total number of base pairs screened by the overall number of mutations identified. Mutation frequency was calculated by dividing the number of identified mutations by the total base pairs screened.

### Sequence analysis tools

The PARSESNP software (project aligned related sequences and evaluate SNPs, Taylor and Greene 2003) was used to show the distribution of mutations within the gene. The higher the PSSM value, the more significant is the impact of the mutation on the function of the encoded protein. A PSSM > 10 is predicted to have a strong influence on the function of the encoded polypeptide. Furthermore, the SIFT tool (sorting intolerant from tolerant, Sim et al. 2012) was used to predict the influence of the mutation on protein function. Scores below 0.05 are predicted to affect protein function. Multiple sequence alignments of full-length protein sequences were performed using the ClustalW2 software (http://www.ebi.ac.uk/Tools/msa/clustalw2/). The 3D structure prediction of the HvSNAC1 protein and its analysed variants with mutations was carried out using ChimeraX 1.9 (Meng et al. 2023; https://www.cgl.ucsf.edu/chimerax/download.html). For homology modeling of the HvSNAC1 structure, the SWISS-MODEL workspace service was utilized (Duvaud et al. 2021; https://www.expasy.org/resources/swiss-model-workspace).

### In silico analysis of cis-element in *HvSNAC1* gene sequence

To identify putative *cis*-elements in the promoter regions of *HvSNAC1*, 2-kb upstream promoter sequences were searched against the PlantCARE database (plant cis-acting regulatory elements, http://bioinformatics.psb.ugent.be/webtools/plantcare/html/) (Lescot et al. 2002).

### Stress treatments and physiological measurements

To evaluate the impact of specific mutations in the *HvSNAC1* gene on the stress response, both mutants and the parent variety ‘Sebastian’, which is the wild-type (WT) for mutants, underwent drought treatment using a protocol extensively detailed by Daszkowska-Golec et al. (2017). The drought treatment involved planting barley seeds in pots filled with a mixture of sandy loam, with sand (w/w; 7:2), and supplemented with a nutrient medium. Initially, barley seeds were germinated in petri dishes containing water-soaked vermiculite and incubated in darkness at 4 °C for 2 days. Subsequently, the germinated seedlings were transferred to a greenhouse for an additional 2 days before being transplanted into the soil, with 15 seedlings per pot. One pot was one biological replicate, and three biological replicates were tested for each genotype and condition. Soil moisture levels were monitored daily throughout the experiment using time-domain reflectometer (TDR) EasyTest equipment provided by the Institute of Agrophysics, Polish Academy of Sciences, Poland. The volumetric water content (vwc) was measured accordingly. The drought assay comprised of two main phases: control growth (CG): seedlings were grown under optimal conditions with soil moisture at 12% vwc for 10 days after transplantation (i). Drought stress (DS): seedlings were subjected to severe drought stress, first soil moisture was gradually reduced from 12 to 3% vwc over 4 days for adaptation to water deficit (AWD), then soil moisture was maintained at 3–1.5% vwc for 10 days (ii). The entire experiment lasted 24 days. During the CG and AWD, seedlings were cultivated in a greenhouse at 20 °C/18 °C with a 16/8-h photoperiod and 400 μEm^−2^ s^−1^ light intensity from high pressure sodium lamps. After AWD, seedlings were transferred to a growth chamber with a temperature regime of 25 °C/20 °C, maintaining the same photoperiod and light intensity as in the greenhouse. The applied drought stress treatment was the same in the initial screening for genotypes carrying mutation in homozygous state as in the main experiment for backcrossed mutants with parent cultivar ‘Sebastian’ followed by identification of plants homozygous for the mutations in F_2_ generation.

### Relative water content (RWC)

RWC was calculated for the detached second leaf of seedlings after 10 days of drought stress (25 days after sowing), according to the formula: RWC (%) = (FW–DW)/ (TW–DW) × 100 (FW–fresh weight; TW–turgid weight after incubation of leaves submerged in distilled water for 24 h in darkness; DW: dry weight after drying leaves at 60 °C for 48 h) (Barrs and Weatherley 1962). Three plants from each of the three pots were used for the RWC analysis, resulting in three biological replicates for each genotype (three plants from each pot represent one biological replicate).

### Gas exchange parameters

Stomatal conductance (µmol H_2_O m^−2^ s^−1^), transpiration rate (mmol H_2_O m^−2^ s^−1^) and photosynthesis rate (µmol CO_2_ m^−2^ s^−1^) of parent cultivar ‘Sebastian’ and two mutants (*hvsnac1.d*, *hvsnac1.e*) in control condition, and after 10 days of drought treatment were measured with Targas-1 (PP Systems, MA, USA). Measurements were performed in three biological replicates, each consisting of the second leaf of the nine plants.

### Analysis of chlorophyll *a* fluorescence

Chlorophyll *a* fluorescence was measured using a plant efficiency analyzer (PocketPEA fluorimeter; Hansatech Instruments Ltd., England). Measurements were performed in three biological replicates, each consisting of the second leaf of the nine plants. Chlorophyll *a* fluorescence was measured in well-watered and drought-treated plants. Before the measurements, the leaves were dark-adapted for 30 min. Measurements were performed in three biological replicates, each consisting of the second leaf of the three plants.

### Chlorophyll, flavonols, anthocyanins contents and NBI index

The chlorophyll meter Dualex Scientific + TM (Force-A, France) was used to determine the chlorophyll, flavonol and anthocyanin contents in the leaves. Dualex also provides the nitrogen balanced index (NBI), which is a good indicator of plant nitrogen status. Measurements were performed in three biological replicates, each consisting of the second leaf of the nine plants.

### ABA spraying

The ABA spray assay was conducted following the methodology outlined in a previous study (Collin et al. 2020). Barley seedlings, including those from WT and both mutants, were cultivated under optimal water conditions in a growth chamber with a temperature regimen of 20 °C/18 °C, under a 16/8-h photoperiod and exposed to a light intensity of 300 μEm^−2^ s^−1^ from fluorescent lamps. The spraying involved treating 10-day-old seedlings with either 5 ml of distilled water (for control) or 200 µM ABA dissolved in distilled water for each plant. Stomatal conductance (µmol H_2_O m^−2^ s^−1^), transpiration rate (mmol H_2_O m^−2^ s^−1^) and photosynthesis rate (µmol CO_2_ m^−2^ s^−1^) were assessed at 30 min, 2, 4 and 6 h post-spray using Targas-1 equipment (PP Systems, MA, USA). Measurements were performed in three biological replicates, each consisting of the second leaf of the nine plants.

### Scanning electron microscopy

For scanning electron microscope (SEM) observations, an approximately 2-cm-long segment from the middle portion of the second leaf was cut off. The segments were immediately immersed in 100% methanol for 4 h at room temperature, dehydrated in 100% ethanol three times for 30 min each and left in 100% ethanol overnight at 4 °C. Small samples (approximately 5 mm × 5 mm) were taken from the fixed and dehydrated segments. The samples were critical point-dried in a Leica EM CPD300 automated critical point dryer (Leica Microsystems, Vienna, Austria) mounted on aluminum stubs with double-sided adhesive carbon tabs, and sputter-coated with gold (15–20-nm-thick film) in a Pelco SC-6 sputter coater (Ted Pella, Inc., Redding, CA, USA). The abaxial epidermis was imaged using a Hitachi SU8010 field emission scanning electron microscope (Hitachi High-Tech Corporation, Tokyo, Japan) at an accelerating voltage of 5 kV.

### Distribution and dimensions of stomata

Stomatal density on the abaxial leaf epidermis (the number of stomata per unit leaf area) was determined from SEM micrographs captured across the entire width of the leaf, avoiding the leaf edges and midrib. Depending on the leaf width, a series of two or three images (each corresponding to an area of 1.2 mm^2^) were captured from the left and right halves of the leaf separated by the midrib. Twelve images were taken for each plant, and the average number of stomata on the surface of 1 mm^2^ was calculated. Three plants were analysed for each genotype and treatment (one plant per box). To measure the stomatal length and width (see Fig. 4E), 16 stomata located midway between the midrib and leaf edge were randomly selected. Measurements were made using the SEM Data Manager software.

### ABA content

The ABA content was assessed from drought experience and control plants following the procedure outlined in prior research (Daszkowska-Golec et al. 2017). Three biological replicates were utilized for each condition (optimal water supply and drought), covering each genotype (WT, *hvsnac1.d* and *hvsnac1.e* mutants). In each biological replicate, the second leaf of one plant was utilized.

### One-hybrid yeast assay

The one-hybrid yeast assay was performed according to the manufacturer’s protocol (Matchmaker® Gold Yeast One-Hybrid Library Screening System User Manual, Clontech). The isolated promoter fragment of *HvPP18* (*protein phosphatase 18*) was 454 bp long and contained two CACG motifs spaced 377 bp apart and was used together with the pAbAi vector to obtain the yeast (*Saccharomyces cerevisiae*) Y1HGold[PP18-AbAi] strain. This gene was selected for analysis because it was experimentally proven that rice SNAC1 can bind to the promoter sequence of *OsPP18* (You et al. 2014b). The selection of yeast colonies with the introduced promoter sequence was performed on medium without uracil (SD/-Ura). A necessary condition for using the yeast one-hybrid assay (Y1H) for this type of analysis is the lack of recognition of the tested promoter sequence by endogenous yeast transcription factors and the inhibition of growth of the transformed yeast construct on a medium containing the antibiotic Aureobasidin A (AbA), that is, no disturbance in the expression of the reporter gene used in this system. For the strain Y1HGold[PP18-AbAi], it was 200 ng/mL AbA. This should be verified experimentally for each tested sequence. To check the protein-DNA interaction, leucine-free medium, SD/-Leu with the antibiotic AbA and a positive control strain Y1Gold[pGADT7-p53/p53-AbAi] were used. For the Y1HGold[OLIGO-AbAi] strain, the concentration of AbA that inhibited growth was 1000 ng/mL. To check the protein-DNA interaction, leucine-free medium, SD/-Leu with the antibiotic AbA and the positive control strain Y1Gold[pGADT7-p53/p53-AbAi] were used.

### RNA-seq study

Transcriptomic analyses were performed as described previously (Daszkowska-Golec et al. 2019). cDNA libraries were prepared for three biological replicates for each condition (optimal water supply and drought) for each genotype (WT, *hvsnac1.d* and *hvsnac1.e* mutants) using second leaf as a tissue for RNA extraction. The RNAqueous™ total RNA isolation kit (ThermoFisher Scientific) was used per the manufacturer protocol, but instead of a lysis buffer, a TriPure isolation reagent (Roche Life Science) was applied. Stranded cDNA library preparation (poly-A selection) and sequencing of 40 M 2 × 150PE (paired-end PE) reads = 12 Gb per sample raw data in fastq were performed in Novogene Company (Hong Kong). For reads mapping, barley genome IBSC_V2 was applied (MorexV2_pseudomolecules_assembly; Mascher et al. 2017; https://nov2020-plants.ensembl.org/Hordeum_vulgare/Info/Index). The selection of differentially expressed genes (DEGs) in our study was based on the following thresholds: *α* = 0.01 with FDR (false discovery rate) adjusted *P*-value and log_2_FC ≥ 1 or ≤  − 1. Enrichment analysis was conducted using the ShinyGO tool (Ge et al. 2020; https://bio.tools/ShinyGO).

### qPCR study

The quantitative real-time RT-PCR (RT-qPCR) technique assessed the genes’ relative expression. The genes were chosen randomly after RNA-seq analysis. Prior to reverse transcription, 1 µg (μg) of total RNA underwent treatment with RNase-free DNase I (Fermentas) for 30 min to eliminate any residual genomic DNA. Subsequently, a RevertAid first strand cDNA synthesis kit (Thermo Scientific) was utilized to synthesize the first-strand cDNA. The resulting cDNA was then diluted 1:5 with ddH_2_O and employed as the template for quantitative PCR.

The qPCR reaction mix, comprising 10 μl, contained 2.5 μl of diluted cDNA, 1 μl of the primer pair mixture (5 μM) and 5 μl of 2 × Master Mix (LightCycler 480 SYBR Green I Master; Roche). The RT-qPCR reactions involved an initial denaturation step at 95 °C for 5 min, followed by 45 cycles of denaturation at 95 °C for 10 s, annealing at 58 °C for 20 s and extension at 72 °C for 10 s. The relative expression level was normalized to a reference gene, *ADP* (*ADP-ribozylation factor 1-like*, Rapacz et al. 2012). Melting curve analysis was done to assess the specificity of qPCR reaction.

Plant material was collected for RNA isolation from each genotype under two conditions: optimal water content and drought. The transcript level of the studied genes was determined using the formula: Ct target gene – Ct reference gene. To assess expression under drought stress, the relative expression of each gene was calculated as the fold change in expression under treatment conditions relative to control conditions for WT using the delta-delta Ct method. Three biological replicates, each comprising one seedling, were utilized for gene expression analysis, with each sample analysed in duplicate technically. The relative expression data were analysed using the LinReq software tool.

### Statistical analyses

Statistical analyses were performed using one-way ANOVA followed by Fisher’s least significant difference (*p* < 0.05) tests to assess differences between treatments and genotypes. All statistical analyses were performed using Dell Statistica (data analysis software system) version 13 (Dell).

## Results

### Identification of mutations in *HvSNAC1* gene using TILLING strategy

**Fig. 1:**
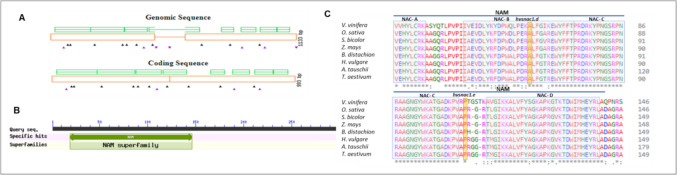
Model of the *HvSNAC1* gene created by PARSESNP tool which shows the distribution as well the type of mutation discovered (**A**), identified NAM domain in HvSNAC1 protein sequence by Conserved Domains Database tool (http://www.ncbi.nlm.nih.gov/cdd) (**B**), and multiple alignment of the SNAC1 homologs partial protein sequences from eight plant species (**C**). Exons: orange boxes, introns: orange lines, conserved domain among plant species: green boxes above the gene structure model. Mutations are indicated as arrowheads below the gene structure model: black—missense mutation; purple—silent, intron or in non-coding sequence-located mutation. Graph C has been generated by the ClustalW2 programme. Identical residues are indicated by stars. The functional domain of the protein: NAM – blue line; NAC subdomain from A to D – grey boxes. NAC subdomains based on Ooka et al. (2003), in yellow are marked positions of substitution in mutants (*hvsnac1.d* and *hvsnac1.e*) subjected to physiological, anatomical and molecular analysis

A TILLING strategy was used to obtain unique research material, a series of alleles of the *HvSNAC1* gene, which were then used to analyse the function of the HvSNAC1 protein. The genomic sequence of *HvSNAC1* (*stress-responsive NAC1*) encoding the NAC-type transcription factor gene is 1499 bp and the encoded protein consists of 330 amino acids (aa) (NCBI: JF796130.1; Ensemble Plants v. MorexV3: HORVU.MOREX.r3.5HG0524540). The TILLING strategy was used to generate and identify mutants of *HvSNAC1* in barley. The analysis was performed with on a 1277-bp amplicon covering the entire open reading frame (ORF) of the gene, and the screening was carried out in 6021 M_2_ plants, giving a total of 7689 kb of analysed sequence. The survey revealed 19 mutations (Table 1), including 16 (84%) located in exons (Fig. 1A). Three different M_2_ plants had the same mutation type (*hvsnac1.f*). Twelve (75%) exon mutations induced a change of aa. The majority of these mutations were GC-to-AT transitions. Forty-two percent of all mutations were found in the homozygous state. The mutation density was one mutation per 405 kb and mutation frequency was 2.5 mutations per 1 Mb.

**Table 1:**
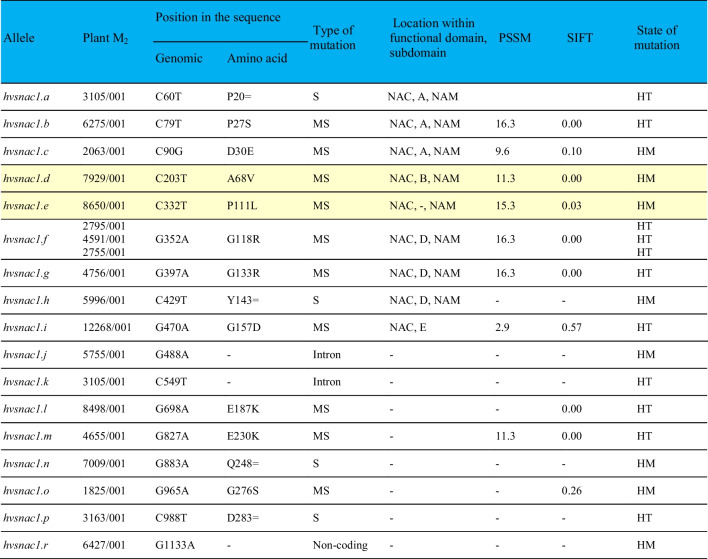
Characteristics of the mutations identified in the *HvSNAC1* gene and their effect on the encoded protein sequence as predicted by the PARSESNP tool

*MS* missense mutation, *S* silent mutation.

The functional domains of the protein: NAM, NAC subdomains from A to E, NAC subdomains based on Ooka et al. (2003)

*PSSM* position-specific scoring matrix, *SIFT* sorting intolerant from tolerant, *HM* homozygous state, *HT* heterozygous state.

Mutants backcrossed with parent cultivar ‘Sebastian’ and subjected to further analysis are marked in yellow.

To characterize protein structure, we performed in silico domain identification by using the Conserved Domains Database tool (http://www.ncbi.nlm.nih.gov/cdd). In the HvSNAC1 protein, NAM (Fig. 1B) and NAC (Fig. 1C) domains were detected. Among all mutations, six and seven missense mutations were identified in the conserved and functionally relevant NAM and NAC domains, respectively (Table 1). In general, these mutations were characterized by high PSSM (position-specific scoring matrix) and low SIFT (sorting intolerant from tolerant) values, indicating that these mutations can have a significant impact on the function of the HvSNAC1 protein. The highest PSSM value was 16.3 for the *hvsnac1.b*, *hvsnac1.f* and *hvsnac1.g* alleles, which carry out the substitution of proline-27 to serine, glicine-118 to arginine and glicine-133 to arginine, respectively. Only in the case of one allele, *hvsnac1.i*, in which the mutation is positioned in the NAC domain but outside of the NAM domain, the PSSM value was low at 2.9 and the SIFT value was high at 0.57. Furthermore, only one mutation detected outside the NAC domain led to a high PSSM value (11.3) for *hvsnac1.m* allele. On the other hand, *hvsnac1.b*, *hvsnac1.c*, *hvsnac1.d*, *hvsnac1.e, hvsnac1.f*, *hvsnac1.g*, *hvsnac1.i* and *hvsnac1.m* carry out substitutions that lead to changes in highly conserved amino acid residues (Table 1). Based on the availability of material and the results of preliminary tests (discussed below), two mutants, *hvsnac1.d* and *hvsnac1.e*, were selected for further physiological, anatomical and molecular analyses. To evaluate the potential impact of mutations in the *HvSNAC1* gene on the encoded protein, the ChimeraX program was used to predict their structures. The substitution of alanine with valine at position 68 (A68V) in the *hvsnac1.d* mutant and proline with leucine at position 111 (P111L) in the *hvsnac1.e* mutant both lead to structural changes in the proteins (Supplementary Materials, Figure S1, S2). However, the structural alterations in *hvsnac1.e* are more pronounced compared to *hvsnac1.d.* As a result, both mutations may impact the functional properties of the HvSNAC1 protein.

### In silico cis-element analysis in the promoter sequence of *HvSNAC1*

An analysis of the *cis*-regulatory sequences in the promoter of the *HvSNAC1* gene was conducted to determine the potential processes and the phytohormones that might regulate the encoded protein’s involvement. To identify putative *cis*-elements in the promoter region of the *HvSNAC1* gene, 2 kb of the sequence upstream relative to the transcription start sites was downloaded from Ensemble Plants v. MorexV3 and was searched against the PlantCARE database. The *cis*-elements that possibly participated in response to light, phytohormones, plant growth and development and stress were identified based on literature and in silico predicted data. A total of 63 *cis*-elements from various categories were identified, with two primary categories emerging: phytohormone (24 elements) and stress-related (22). The next most numerous category was light-related (13), and the least numerous was plant growth and development, where only four related *cis*-elements were identified (Table 2). Among phytohormone-related elements, some may participate in response to abscisic acid (ABA), auxin (AUX), gibberellins (GA) and methyl jasmonate (MeJA) (Table 2). Among the stress-related *cis*-elements, CCAAT-box (MYBHv1 binding site), DREs (dehydration-responsive element), GC-motif, LTRs (low temperature-responsive elements), MBS (MYB binding site), MYC (MYC responsive element) and MYB (MYB responsive element) were detected (Table 2). This suggests that HvSNAC1 might be involved in different stress and hormone response pathways, as well as in light responses and meristem function.

**Table 2:** Identified *cis*-acting elements in promoter sequences of *HvSNAC1*

*cis*-element related to	Motif	Number	Strand	
			+	-
Light	AE-box	1	0	1
	Box 4	1	0	1
	GATA-motif	1	0	1
	G-Box	4	2	2
	GT1-motif	2	0	2
	L-box	1	1	0
	Sp1	1	0	1
	TCT-motif	2	1	1
Phytohormone	ABRE	8	2	6
	CGTCA-motif	6	2	4
	GARE-motif	2	0	2
	TGA-box	2	1	1
	TGACG-motif	6	4	2
Plant growth and development	CAT-box	2	0	2
	CCGTCC-motif	2	1	1
Stress	CCAAT-box	1	1	0
	DRE	3	1	2
	GC-motif	2	2	0
	LTR	2	1	1
	MBS	4	4	0
	MYC	2	1	1
	MYB	8	7	1

Phytohormone-responsive *cis*-elements: ABRE – ABA responsive element; CGTCA – methyl jasmonate responsive element, GARE – gibberellin responsive element, TGA – auxin responsive element, TGACG – methyl jasmonate responsive element; plant growth and development: CAT-box **–** related to meristem expression, CCGTCC-motif, meristem specific activation; stress-responsive *cis*-elements: CCAAT-box – MYBHv1 binding site; DRE – dehydration responsive elements; GC-motif– anoxic responsive element; LTR – low temperature responsive element, MBS – MYB binding sites, MYC – MYC responsive element, MYB responsive element (MYB recognition site and MYB like sequence).

### Characteristics of mutants carrying changes in *HvSNAC1* gene under drought and exogenous treatment with ABA

To determine whether the induced mutations affect the functions of the HvSNAC1 protein, an analysis of mutants was conducted under various conditions (drought stress and ABA spraying) and parameters (relative water content in leaves, RWC; gas exchange; density and morphology of stomata; nitrogen balance index). In the first phase, an initial screening was performed for RWC, a basic parameter used to assess drought stress tolerance, for seven alleles carrying mutations in the homozygous state (Supplementary Materials, SFigure 3). Previous studies in rice demonstrated role of SNAC1 in regulating stomatal closure during drought stress, resulting in higher leaf water content in forms with overexpression of this gene. Our goal was to expand knowledge on functions of this protein in response to drought stress and to the ABA depend pathway. Promoter analysis of the *HvSNAC1* gene revealed the presence of *cis*-regulatory elements characteristic for ABA response in barley.

To learn more about the function of HvSNAC1, we selected two alleles from the analysed pool that showed higher leaf water content in preliminary studies, had the highest PSSM and possessed mutations in the NAC domain and subjected them to detailed analysis. First, we performed backcrossing of these alleles with the parent variety ‘Sebastian’ and plants homozygous for the mutations were identified in F_2_ generation. We then examined the response of these mutants at the physiological level (gas exchange during drought stress and ABA spraying to reveal potential differences in stomatal movements; ABA content in leaves during drought stress; and nitrogen balance index) and anatomical level (stomatal density). We analysed the NBI parameter because studies in rice have shown that SNAC1 regulates the expression of *OsNRT2.1* (*nitrate transporter 2.1*). Additionally, we conducted an analysis of chlorophyll, flavonol and anthocyanin contents, as their levels can influence the process of photosynthesis and photosynthetic efficiency itself. So far, SNAC1’s involvement in regulating this process has not been demonstrated.

Two mutants, *hvsnac1.d* and *hvsnac1.e* selected as previously mentioned, were exposed to drought stress together with their parent cultivar ‘Sebastian’ (wild-type, WT) to check if the barley *HvSNAC1* gene functions in the drought stress response. The *hvsnac1.d* mutant carries a substitution (A68V) in the B subdomain of the NAC protein, while *hvsnac1.e* has a substitution (P111L) between the C and D subdomains (Fig. 1).

**Fig. 2:**
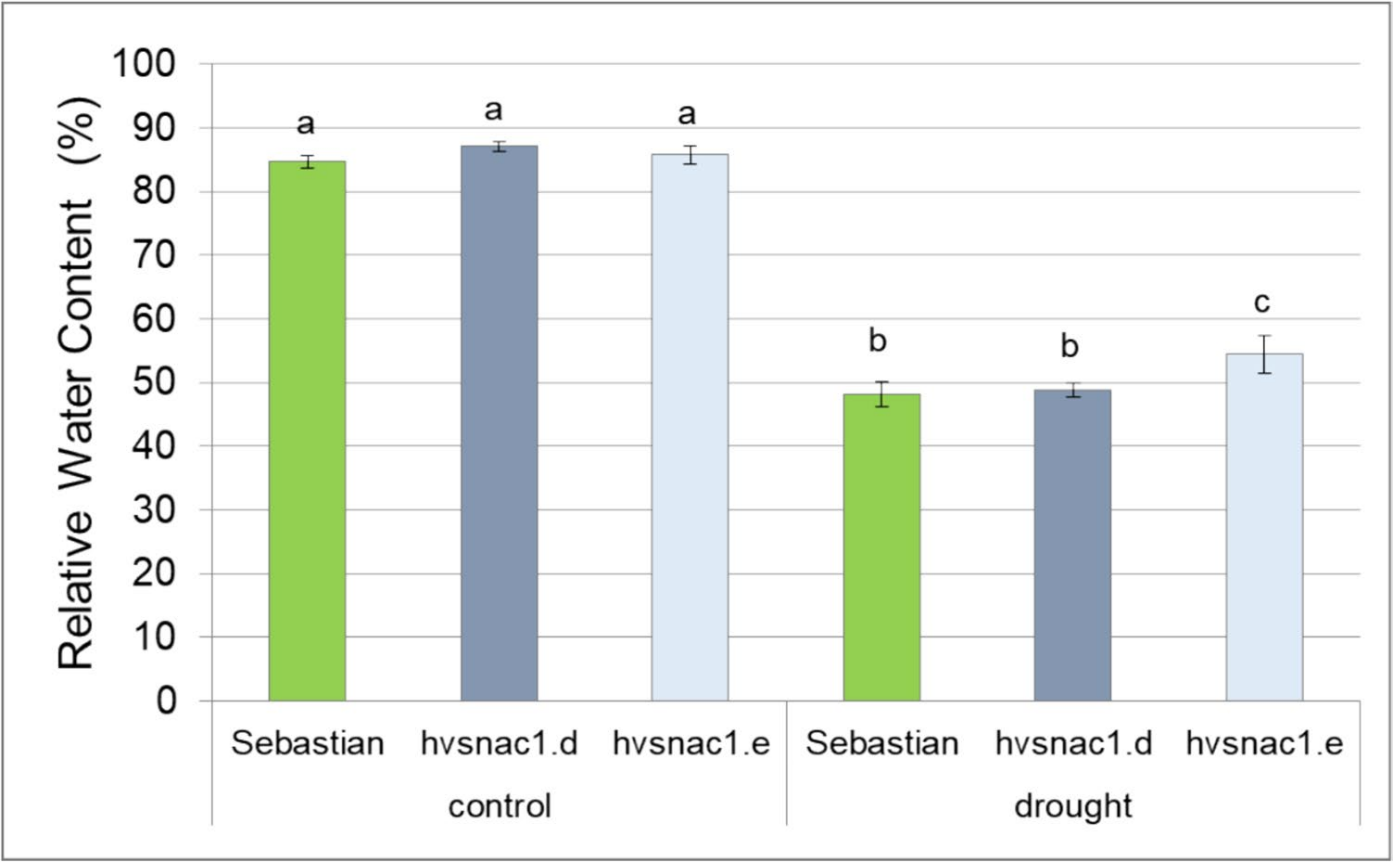
Relative water content (RWC in %) of parent cultivar ‘Sebastian’ and mutants: *hvsnac1.d* and *hvsnac1.e* in control condition, and after 10 days of drought treatment. The values are presented as the means ± SE of nine plants per one biological replication, and three biological replications were used. Statistically significant differences between different genotypes and growth conditions were assessed using a one-way ANOVA followed by the Fisher least significant difference (LSD) test (*p* < 0.05) and are indicated by different letters

The 15 days old barley seedlings were exposed to 10 days of water deficiency or grown for the same time under optimal water conditions. At the end of this period, we measured relative water content (RWC) in leaves, a relevant indicator of stress tolerance. In the control condition, there were no significant differences among all analysed genotypes (Fig. 2). Under drought stress conditions, all tested genotypes presented strongly decreased RWC (Fig. 2). This indicates that the stress condition was severe (Supplementary Materials SFigure 4). Only one mutant, *hvsnac1.e*, showed differences comparing to the parent cultivar ‘Sebastian’ in RWC level under drought stress and stored 6% more water in leaves (Fig. 2).

Additionally, we conducted a post-harvest analysis of plants grown under controlled conditions for ‘Sebastian’ and its mutants, measuring the following parameters: stem length, spike length, awn length, number of tillers, number of grains per plant and grain weight per plant. Among these traits, only the number of tillers remained unchanged across the analysed genotypes. Stem length, awn length and the number of grains per plant were reduced in *hvsnac1.e* compared to WT. Spike length and grain weight per plant were decreased in both mutants compared to WT (Supplementary Materials, SFigure 5).

**Fig. 3:**
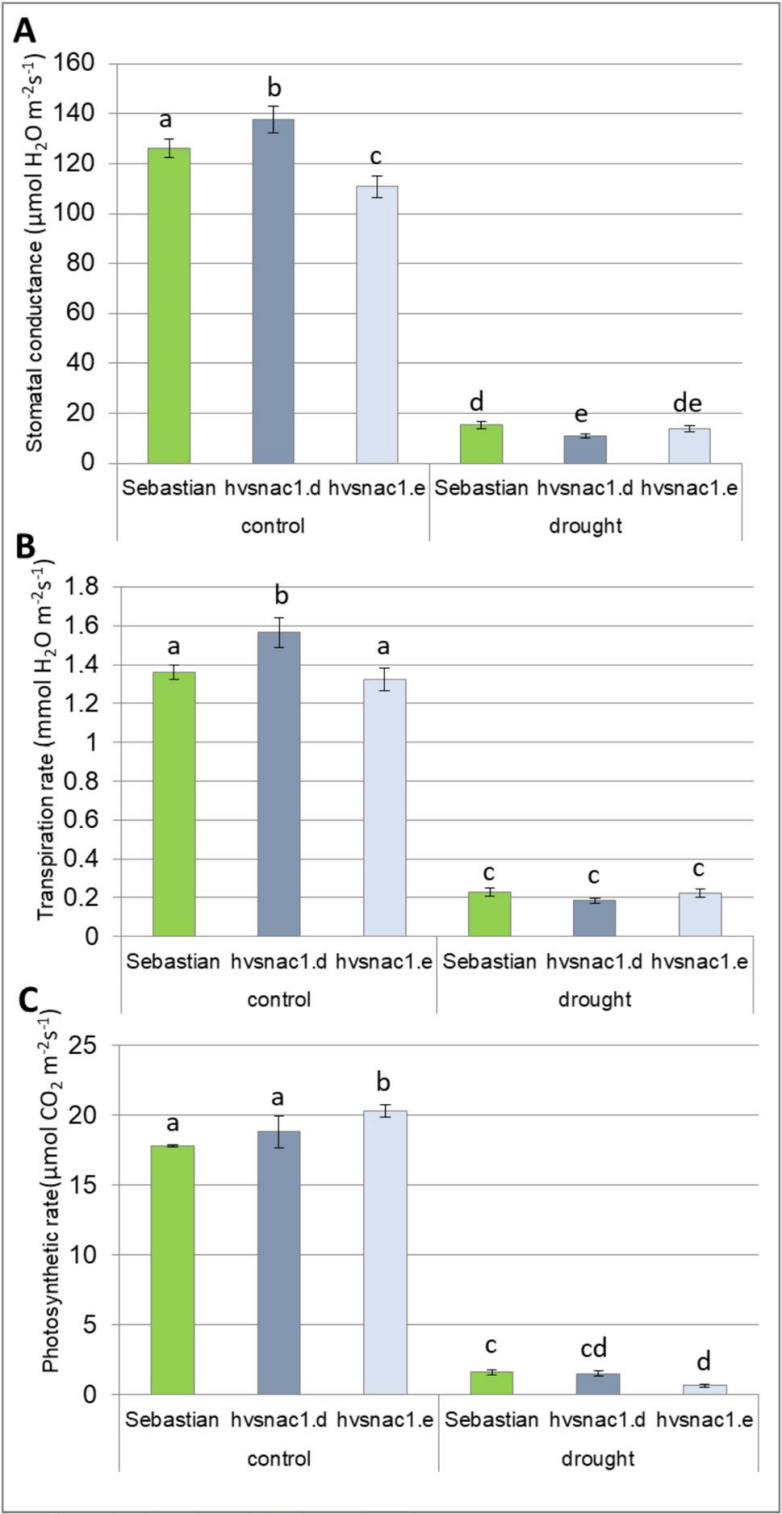
Stomatal conductance (**A**), transpiration rate (**B**) and photosynthesis rate (**C**) of parent cultivar ‘Sebastian’ and two mutants (*hvsnac1.d, hvsnac1.e*) in the control condition, and after 10 days of drought treatment. The values are presented as the means ± SE of nine plants per one biological replication, and three biological replications were used. Statistically significant differences between different genotypes and growth conditions were assessed using a one-way ANOVA followed by the Fisher least significant difference (LSD) test (*p* < 0.05) and are indicated by different letters

Stomatal conductance measures the degree of stomatal opening and can be used as an indicator of plant water status. For this reason, stomatal conductance was measured under both optimal water supply and drought stress for the WT and the two mutants. We found minor differences in stomatal conductance among genotypes under optimal water supply, *hvsnac1.d* presented higher and *hvsnac1.e* lower stomatal conductance than the WT (Fig. 3A). After 10 days of drought stress, all genotypes analysed displayed a significant reduction in stomatal conductance. The *hvsnac1.e* mutant displayed no differences compared to ‘Sebastian’ variety, whereas *hvsnac1.d* showed lower values of stomatal conductance compared to ‘Sebastian’ (Fig. 3A). So, we may conclude that the higher RWC (6%) of *hvsnac1.e* compared to ‘Sebastian’ variety observed after 10 days of drought stress is not caused by differences in stomatal conductance in this specific time of stress treatment.

Ten days of drought stress led to a significant decrease in the rate of transpiration (Fig. 3B) and photosynthesis (Fig. 3C) in all the genotypes analysed. Under optimal water supply conditions, the transpiration rate was higher only in *hvsnac1.d* mutant comparing to the WT (Fig. 3B), whereas the photosynthesis rate was higher in *hvsnac1.e* (Fig. 3C). In turn, the *hvsnac1.d*, which showed lower stomatal conductance than the ‘Sebastian’ variety after 10 days of drought stress (Fig. 3A), exhibited no differences in both transpiration rates (Fig. 3B) and in photosynthesis rate compared with the parent cultivar (Fig. 3C). While, *hvsnac1.e* showed lower photosynthesis rate comparing with ‘Sebastian’ variety after 10 days of drought stress (Fig. 3C). After 10 days of drought stress, *hvsnac1.e* showed no differences with parent cultivar ‘Sebastian’ in the case of stomatal conductance and transpiration rate. These parameters do not explain the higher RWC observed in this mutant after stress treatment (Fig. 3A, B).

### Density and size of stomata under drought stress

**Fig. 4:**
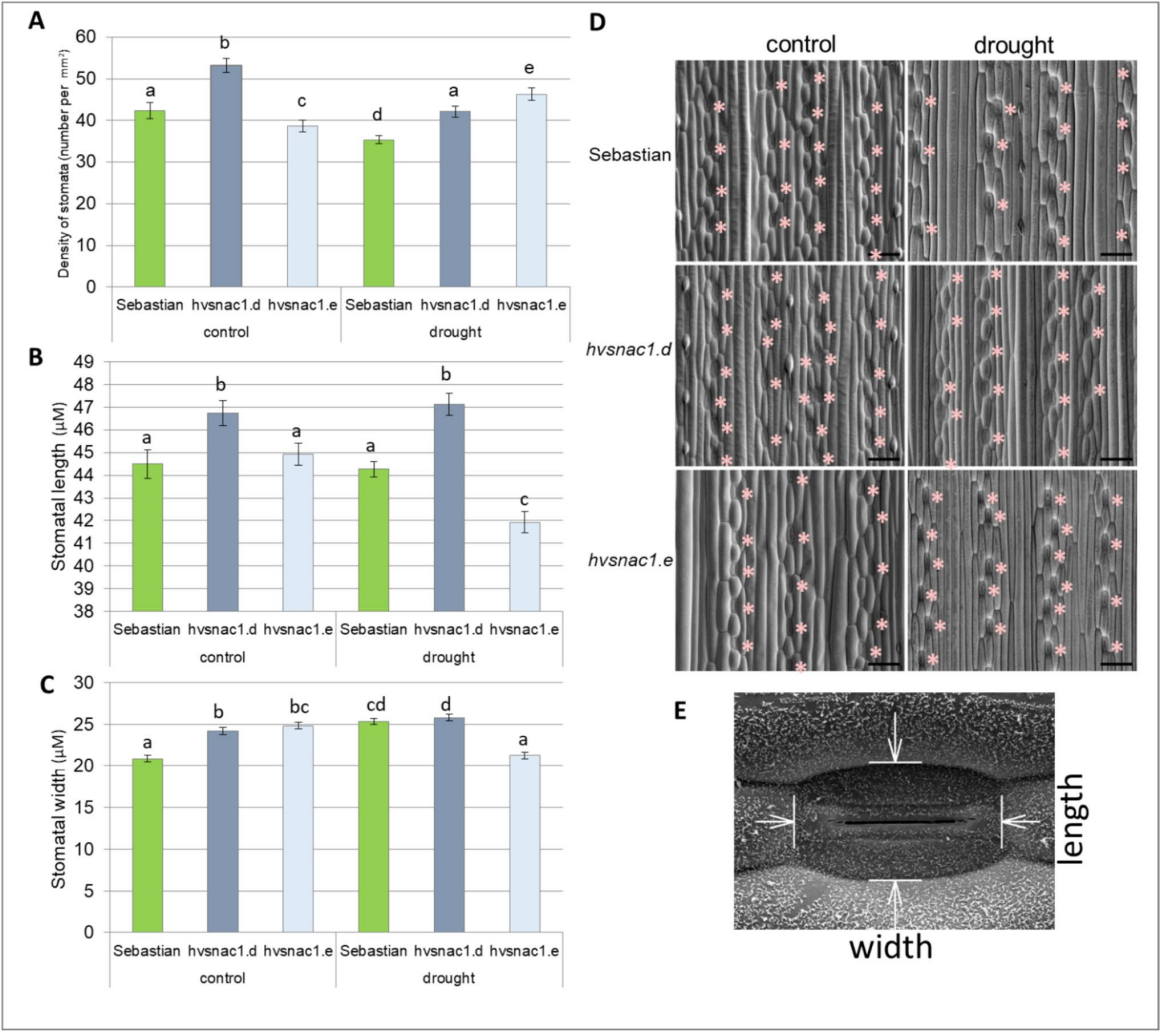
The abaxial stomatal density (number per mm^2^) (**A**), stomatal length (**B**) and width (**C**) (µm), scanning electron microscopy (SEM) images of stomata distribution on the abaxial leaf surface (**D**), determination of stomata dimensions (**E**) of parent cultivar ‘Sebastian’ and mutants: *hvsnac1.d*, *hvsnac1.e* in control conditions and after 10 days of drought treatment. The values are presented as the means ± SE of nine plants per one biological replication, and three biological replications were used. Statistically significant differences between different genotypes and growth conditions were assessed using a one-way ANOVA followed by the Fisher least significant difference (LSD) test (*p* < 0.05) and are indicated by different letters. Scale bar in (**D**): 100 μm. Stomata are marked by asterisks

In addition, we checked the density and size of stomata on the second leaf on the abaxial side of all genotypes analysed under both optimal water supply and after 10 days of severe drought stress. Both mutants exhibited differences in stomata density in control conditions compared with ‘Sebastian’, *hvsnac1.d* displayed a higher, while *hvsnac1.e* a lower stomatal density (Fig. 4A). After 10 days of drought stress, a reduction in the number of stomata was observed both in ‘Sebastian’ and *hvsnac1.d* mutant compared with control conditions. At the same time, in *hvsnac1.e* mutant, an increase in this value was observed (Fig. 4A). Both mutants *hvsnac1.d* and *hvsnac1.e* displayed a higher density of stomata compared with ‘Sebastian’ variety under drought stress (Fig. 4A). Moreover, differences in stomatal morphology in these mutants have been detected. The *hvsnac1.d* presents higher stomatal length both under optimal water supply and drought compared with ‘Sebastian’ variety (Fig. 4B), and higher stomatal density compared with ‘Sebastian’ variety under drought (Fig. 4A), but lower stomatal conductance in this condition (Fig. 3B). This indicates that the lower stomatal conductance in *hvsnac1.d* under drought stress could result from changes in stomatal morphology or stomatal regulation.

In the case of *hvsnac1.e*, an increase in stomatal density was detected when the control and drought stress treatments were compared (Fig. 4A). Interestingly, differences in stomatal size in *hvsnac1.e* compared with the parent variety ‘Sebastian’ under both control and drought stress were detected (Fig. 4BC). The stomata were wider in *hvsnac1.e* compared with ‘Sebastian’ under optimal water supply in contrast to drought stress, where both the length and width of stomata were lower (Fig. 4BC). The higher RWC of *hvsnac1.e* compared to WT observed after 10 days of drought stress is rather not connected with differences in stomatal density and morphology between mutant and ‘Sebastian’. This mutant displayed higher stomatal density under drought stress compared with WT, what potentially could affect the stomatal conductance, but simultaneously the size of stomata was smaller, both length and width what could offset the density effect (Fig. 4A, B, C). A smaller size of stomata could influence the total pore area, potentially resulting in a changed gas exchange.

### Exogenous ABA treatment and ABA content in leaf tissue

**Fig. 5:**
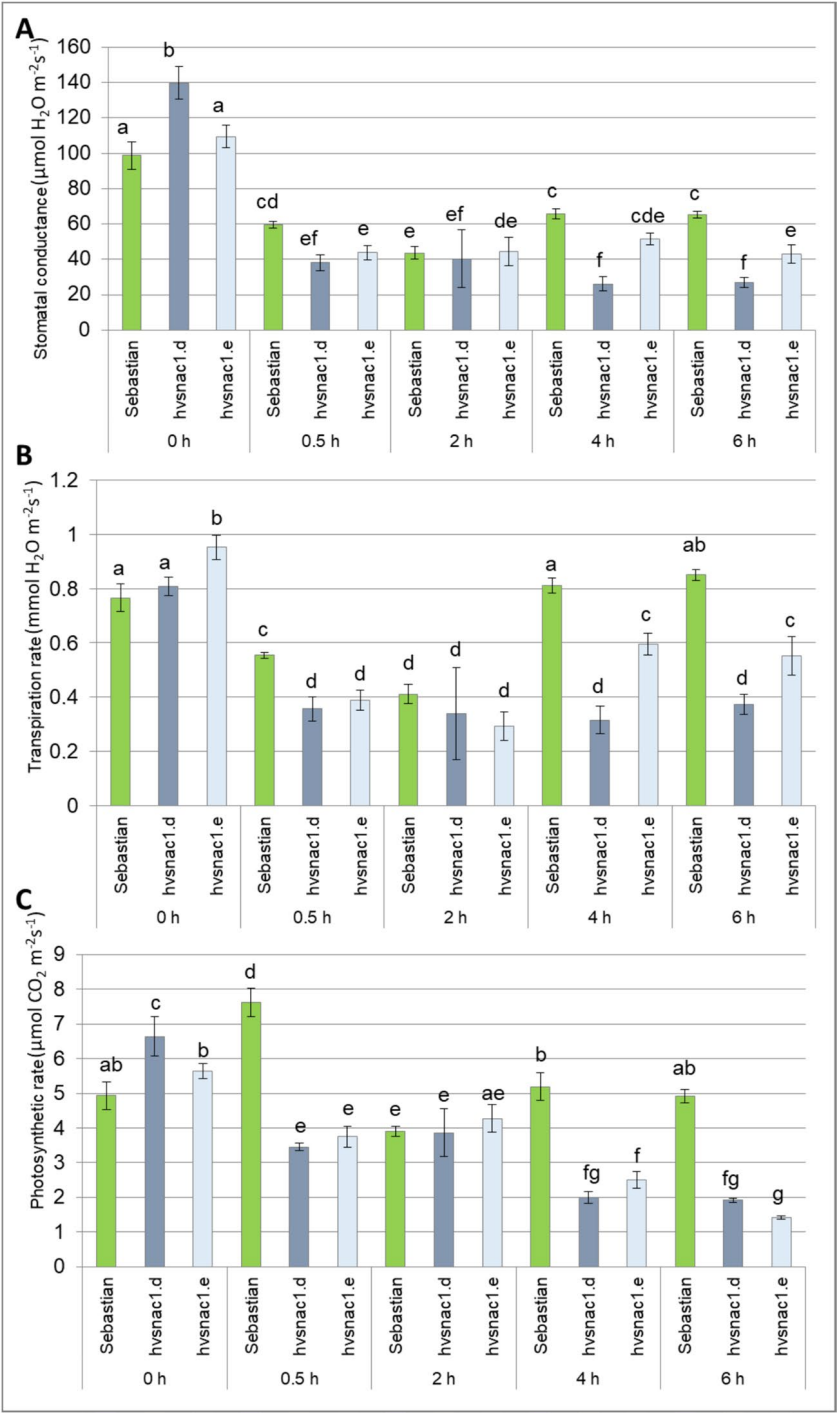
Stomatal conductance (**A**), transpiration rate (**B**) and photosynthesis rate (**C**) of parent cultivar ‘Sebastian’ and two mutants (*hvsnac1.c*, *hvsnac1.d*) in control condition, and after 30 min, 2, 4 and 6 h after ABA spraying. The values are presented as the means ± SE of nine plants per one biological replication, and three biological replications were used. Statistically significant differences between different genotypes and growth conditions were assessed using a one-way ANOVA followed by the Fisher least significant difference (LSD) test (*p* < 0.05) and are indicated by different letters

Based on in silico analysis of the promoter region of *HvSNAC*1, we assumed that its expression might be regulated by the ABA-induced signaling pathway (Table 2). We wanted to check if mutants in this gene have different sensitivities to ABA-induced stomatal movement compared to WT. We assumed that changed sensitivity and faster stomatal responses could lead to a more efficient uptake of CO_2_ during stomatal opening and as small as possible water loss during stomatal closure. Plants with highly responsive stomata might have a higher water content in their leaves. To test whether stomata in the mutants are sensitive to exogenously applied ABA, we measured stomatal conductance 30 min, 2, 4 and 6 h after ABA spraying (Fig. 5A). The response of all analysed stomata to ABA was observed 30 min after treatment. However, this treatment time caused a higher reduction in stomatal conductance of both mutants than WT, which further indicated faster stomata closure. After 2 h of ABA treatment, all analysed genotypes presented the same level of stomatal conductance, which resulted in further stomatal closure in the ‘Sebastian’ variety. Further extension of time from ABA spraying till 4 h did not cause any additional reduction in the stomatal conductance of the mutants, but the stomatal conductance of WT returned to the level observed after 2 h of treatment. Noticeably, varying sensitivity of the analysed stomata genotypes to ABA became apparent after 6 h of treatment. Both mutants showed no change in stomatal conductance from 30 min till 6 h after ABA spraying (Fig. 5A). These results indicate that the stomata of both mutants were more sensitive to ABA than the WT during stomatal closure. Interestingly, we detected an impairment in the stomatal reopening of the mutants. The stomata of WT plants opened faster than those of mutants. Under the same ABA treatment, the transpiration and photosynthesis rates in WT and both mutants have been measured (Fig. 5B, C). In general, changes in these parameters correspond to those observed for stomatal conductance (Fig. 5A).

**Fig. 6:**
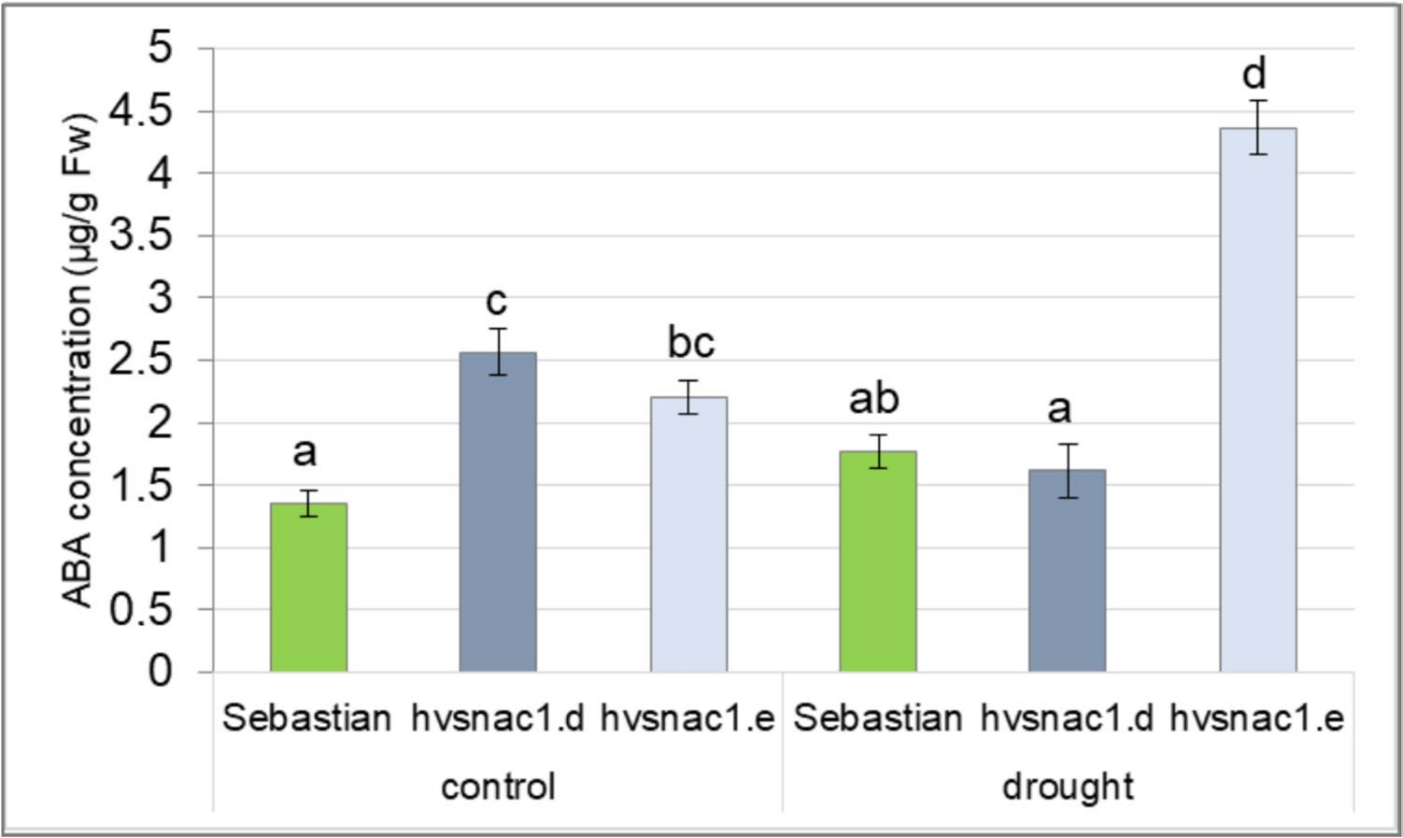
ABA content in leaf tissue of parent cultivar ‘Sebastian’ and mutants (*hvsnac1.d*, *hvsnac1.e*) in control condition and after 10 days of drought stress. The values are presented as the means ± SE, one plant per one biological replication, three biological replications were used. Statistically significant differences between different genotypes and growth conditions were assessed using a one-way ANOVA followed by the Fisher least significant difference (LSD) test (*p* < 0.05) and are indicated by different letters

Taking into account that we observed differences in ABA-induced stomatal movement among the analysed mutants and WT, we aimed to test whether these genotypes exhibit any alterations in ABA content. The concentration was measured in the leaf tissue of non-stressed plants and after 10 days of drought stress. Both mutants presented higher concentration of ABA in control conditions compared to the WT (Fig. 6). After the stress condition, the ABA level increased compared with the control condition only in *hvsnac1.e* mutant by 68% more than in ‘Sebastian’ variety (Fig. 6). To summarize the most important findings for this mutant, it showed more sensitivity to ABA-induced stomatal movement phenotype, faster and long-lasting of stomatal closure after 6 h from ABA spraying (Fig. 5A), displayed lower photosynthetic rate under drought stress (Fig. 3C), higher RWC in leaf after drought, as well as changes in stomatal density (Fig. 4A) and morphology (Fig. 4B, C) compared with WT.

### Photosynthesis efficiency

We also performed a chlorophyll *a* fluorescence (ChIF) analysis to get more in-depth information on photosynthesis efficiency under drought stress in the genotype of study. We analysed the effect of 10 days of drought stress on the photosynthetic efficiency of barley mutants (*hvsnac1.d*, *hvsnac1.e*) and WT. We analysed the selected parameters associated with photosynthesis efficiency in barley leaves: PI_ABS_ (performance index per absorption), *F*_*v*_/*F*_*m*_ (maximum quantum yield of photosystem II), ABS/CS_0_ (energy absorbed per excited cross-section), TR_0_/CS_0_ (energy trapped per CS), ET_0_/CS_0_ (electron transport per CS), DI_0_/CS_0_ (energy of dissipation per CS), RE_0_/CS_0_ (electron flux reducing end electron acceptors at the PSI acceptor side) and RC/CS_0_ (density of active reaction centers per CS) (STable 1). PI_ABS_ provides quantitative information on the general state of plants and their vitality. Drought stress led to a massive decrease of this parameter in all analysed genotypes, from 30 to 49% for *hvsnac1.e* and *hvsnac1.d*, respectively. Other parameters in which changes in all genotypes after drought stress have been detected were *F*_*v*_/*F*_*m*_, RC/CS_0_, ABS/CS_0_ and DI_0_/CS_0_ (Supplementary Materials, STable 1). Only the TR_0_/CS_0_ was unchanged between the analysed conditions and genotypes. The most interesting parameters were ET_0_/CS_0_ and RE_0_/CS_0_, where differences between ‘Sebastian’ and *hvsnac1.e* under drought stress were detected. Additionally, for RE_0_/CS_0_, differences between these genotypes under control conditions were noticed. Interestingly, for *hvsnac1.e*, no decrease in either ET_0_/CS_0_ or RE_0_/CS_0_ was detected between the control and drought conditions. This indicates higher dynamics of electron transport within photosystems in *hvsnac1.e* compared with WT, which corresponds with higher RWC in the leaves (Fig. 2).

Taken together, these results indicate that, in general, drought stress leads to decreased photosynthetic efficiency in all analysed genotypes. Some differences between the responses of *hvsnac1.e* and the ‘Sebastian’ cultivar to drought stress have been detected. The *hvsnac1.e* presented higher values of electron transport per CS (ET_0_/CS_0_) and electron flux reducing end electron acceptors at the photosystem I (PSI) acceptor side (RE_0_/CS_0_) compared with WT (Table 3), what suggests higher dynamics of electron transport within photosystems in the mutant.

**Table 3:** Drought-responsive DEGs, downregulated and specific only for mutants under 10 days drought stress

No	Acc. Num	Annotation	Altered expression level (log2 FC)		Arabidopsis homolog	Gene name	Expressed in guard cell	Number of CACG motif
			*hvsnac1.e*	*hvsnac1.d*				
1	HORVU1Hr1G075570	Profilin	−2,1	−4,5	AT2G19770	PRO4	no	1
2	HORVU4Hr1G046610	Heavy metal-associated isoprenylated plant protein 32	−2,2	−1,5	-	-	-	8
3	HORVU5Hr1G107240	Putative metal-nicotianamine transporter YSL3	−2,5	−2,7	AT1G48370	YSL8	yes	1
4	HORVU2Hr1G118700	EMBRYO SURROUNDING FACTOR 1-like protein	−2,4	−2,4	AT4G21720	Defensin-like protein	yes	1
5	HORVU7Hr1G114400	Putative receptor-like protein kinase	−3,0	−2,4	AT1G80640	Protein kinase superfamily	yes	12
6	HORVU2Hr1G013500	Arogenate dehydratase/prephenate dehydratase 2 chloroplastic	−2,0	−2,1	AT3G07630	ADT2	yes	1
7	HORVU6Hr1G033190	Ubiquitin-conjugating enzyme E2 22-like	−2,6	−2,5	-	-	-	3
8	HORVU5Hr1G103990	Chemocyanin	−2,4	−2,3	-	-	-	3
9	HORVU3Hr1G075210	Expansin	−5,6	−7,6	AT2G40610	EXPA8	yes	5
10	HORVU0Hr1G017960		−3,8	−3,9	-	-	-	3
11	HORVU7Hr1G098260	Xyloglucan endotransglucosylase/hydrolase	−4,6	−3,3	-	-	-	4
12	HORVU5Hr1G098920	Pectin acetylesterase	−2,4	−4,2	AT5G23870	PAE9	yes	3
13	HORVU3Hr1G068650	P-glycoprotein homologue	−3,6	−2,2	-	-	-	7
14	HORVU6Hr1G012270	Putative terpene synthase	−3,8	−3,4	AT1G61680	TPS14	yes	7

2 kb promoter sequences were investigated for CACG-motif identification.

### Content of chlorophyll, flavanol, anthocyanins and nitrogen balance index contents

Next, we checked the content of chlorophyll, flavanol, anthocyanins, and nitrogen balance index (NBI) in the mutants and WT in control and drought stress conditions as important factors that potentially influence photosynthesis performance. Drought stress led to a decrease in chlorophyll content and NBI index in all analysed genotypes compared with the control condition, except for the flavonoid and anthocyanin indices, which increased after drought treatment. No differences between the analysed genotypes in both control conditions and drought stress in all parameters except the NBI index were detected (Supplementary Materials, SFigure 6). *Hvsnac1.d* presented a lower NBI index than WT under drought stress.

### One-hybrid yeast assay to confirm the *cis*-regulatory motif for native HvSNAC1

**Fig. 7:**
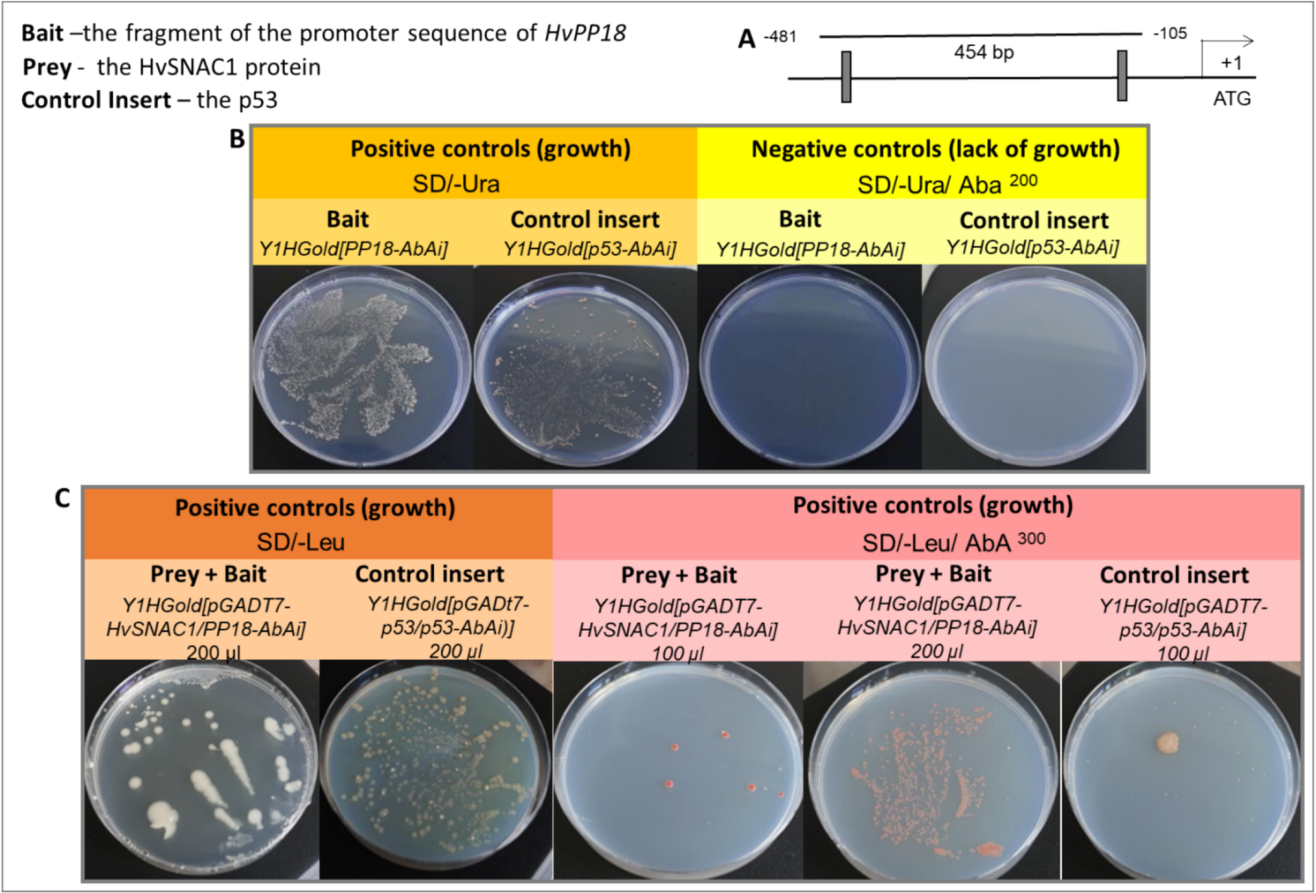
*HvPP18* is directly regulated by HvSNAC1. **A** Diagram of the *HvPP18* promoter showing the DNA fragments was used in the yeast one-hybrid assay. Black rectangles indicate the CACG motifs within the promoter sequence of *HvPP18*. **B** Screening in SD/-Ura (synthetic defined (SD) yeast medium without uracil) containing ABA (aureobasidin A). **C** Screening in SD/-Leu medium (without leucine) containing or not containing ABA

The conducted analysis aims to identify the *cis*-regulatory sequence for HvSNAC1 in barley, which we then utilized to identify potential targets of HvSNAC1 identified after global transcriptome analysis. We used a one-hybrid yeast assay to confirm that the NACRS (NAC recognized sequence)–CACG sequence, which was found as a target that is bound by rice homolog SNAC1 in vitro (You et al. 2014a, b), is also a *cis*-regulatory motif in barley genes potentially regulated by HvSNAC1. To test whether HvSNAC1 can bind to this sequence, we chose the promoter of *HvPP18* (*protein phosphatase 18*) gene, which contains two CACG sequences located upstream of the start codon (Fig. 7A). We confirmed that HvSNAC1 binds in vitro to the tested promoter fragment; therefore, HvSNAC1 may be a potential regulator of *HvPP18* transcription (Fig. 7A, B). Based on the results of these analyses, an artificial promoter sequence was designed: an oligonucleotide 97 bp in length, which also contained two CACG motifs spaced by 72 bp. Also, in this case, HvSNAC1 binds in vitro to the tested fragment. These results suggested that the barley homolog of rice SNAC1 could bind to the CACG sequence in vitro and at the same time; it was shown that this motif serves as a *cis*-regulatory sequence for HvSNAC1 in barley.

### Transcriptome analysis of mutants carrying changes in *HvSNAC1* gene using RNA-seq under drought stress

Global transcriptome analysis was conducted for two mutants and WT during drought stress. Initially, differentially expressed genes (DEGs) between drought and optimal irrigation conditions were identified for each genotype. Then, from this pool of genes, those specific only for the mutants were identified. We are searching for new potential target genes for HvSNAC1, and we expect that the induced mutations will influence its function. Of particular interest to us was the group with specifically reduced gene expression level in mutants, and associated with the regulation of stomatal movements and density, as differences in these traits were identified during the phenotypic analysis of mutants.

To explore the impact of mutations in *HvSNAC1* gene on drought response on molecular level, we performed differential expression analysis using RNA-seq. The RNA was extracted from tissue collected within the same experiment and simultaneously for both mutants and WT, but sequencing was performed separately. Considering results obtained after the phenotypic characterization of mutant, we concentrated on genes that might regulate stomatal dynamics under drought. Principal component analysis (PCA) of the obtained RNA-seq data revealed significant differentiation between the WT and the mutants (*hvsnac1.e* and *hvsnac1.d*) in both control and drought conditions (Supplementary Materials, SFigure 7). Biological replicates from the same genotype and condition generally clustered together, with only one exception for a single replicate. PC1 accounted for a large proportion of the variability, explaining 51.04% in the analysis of *hvsnac1.e* and ‘Sebastian’, and 54.53% in the analysis of *hvsnac1.d* and ‘Sebastian’.

qPCR analysis revealed that drought stress led to a 16-fold increase in the expression level of the *HvSNAC1* gene compared to control conditions in the ‘Sebastian’ variety. A similar expression profile was observed in the mutants, with a 15-fold and sevenfold increase in *hvsnac1.d* and *hvsnac1.e*, respectively (Supplementary Materials, SFigure 8).

**Fig. 8:**
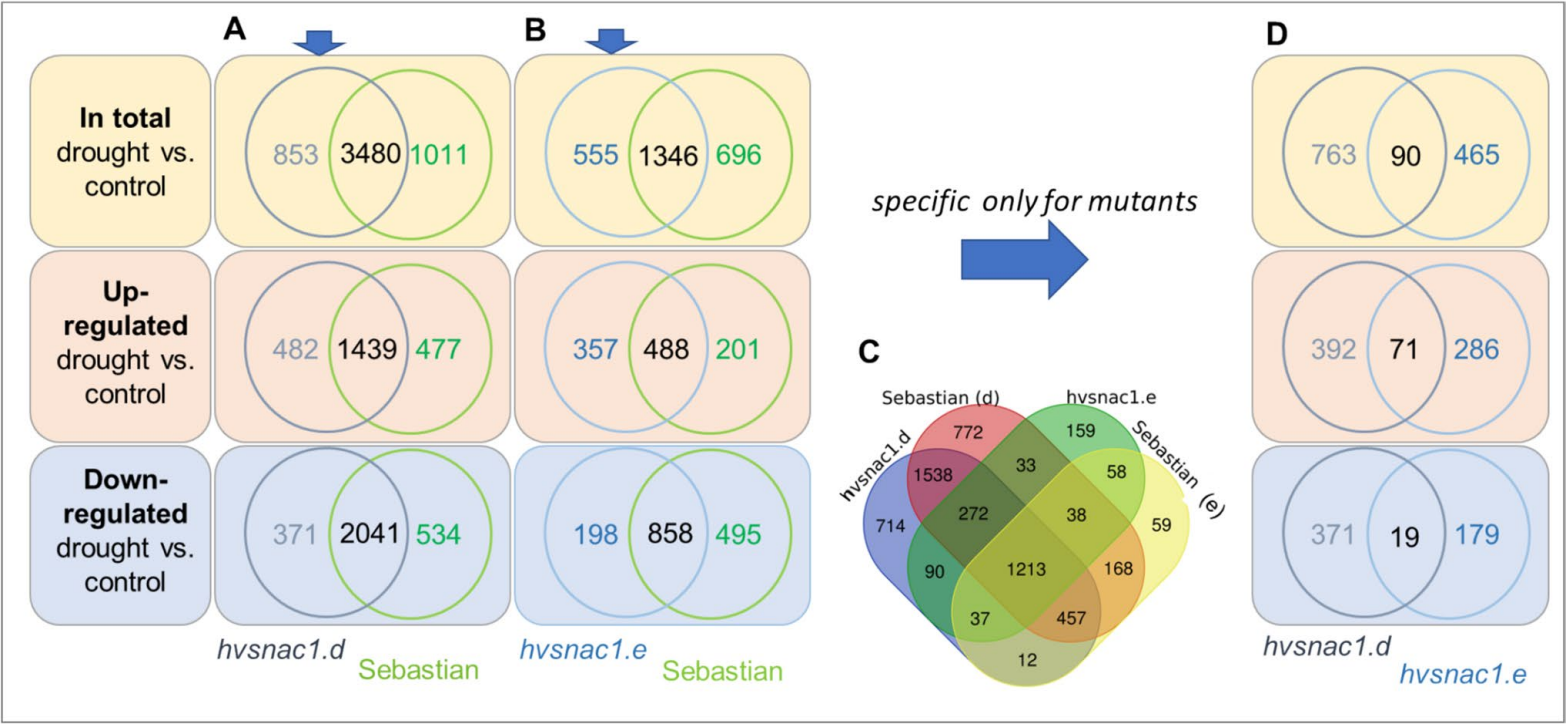
The number of differentially expressed genes (DEGs) in the transcriptome of barley leaves in *hvsnac1.d* and ‘Sebastian’ parent variety (**A**), *hvsnac1.e* and ‘Sebastian’ (**B**), both mutants and ‘Sebastian’ from each analysis (**C**), *hvsnac1.d* and *hvsnac1.e* (**D**). DEGs were determined separately for each compared genotypes for plants grown 10 days in severe drought condition compared to optimal water supply growth condition. DEGs were identified under *α* = 0.01 with FDR adjusted *P*-value and log_2_FC ≥ 1 or ≤  − 1

In *hvsnac1.d*, we identified 4333 differentially expressed genes (DEGs) in the leaves of plants grown for 10 days under severe drought conditions when compared to those grown under optimal water supply (Supplementary Materials, STable 2). In the parent cultivar, ‘Sebastian’, 4491 DEGs were detected (Supplementary Materials, STable 2). Among these genes, 3480 were common for the mutant and ‘Sebastian’. DEGs specific for the mutant—853 genes and ‘Sebastian’—1011 genes were also observed (Fig. 8A). In the second mutant, *hvsnac1.e*, 1901 DEGs were identified by the same comparison as for the previous mutant (Supplementary Materials, STable 3). In turn, in parent cultivar ‘Sebastian’, 2042 DEGs were detected after exposure to drought stress (Supplementary Materials, STable 3). Among these genes, 1346 genes were identified as common for the WT and the mutant. Genes specific for the mutant—555 genes and ‘Sebastian’—696 genes were also observed (Fig. 8B). For selected DEGs like *HvPLA2* (*phospholipase A2-alpha,* HORVU4Hr1G061740), *HvNLP2* (*plant regulator RWP-RK family protein*, HORVU3Hr1G032170), *HvCRY2* (*cryptochrome-2*, HORVU6Hr1G058730) and *HvLHCB* (*chlorophyll a-b binding protein*, HORVU6HR1G091650), we performed the analysis of expression level using RT-PCR, and the results obtained after RNA-seq and RT-qPCR analysis are consistent (Supplementary Materials, SFigure 9).

Among the drought-responsive DEGs common to all investigated genotypes, some drought marker genes were identified. Among them was the *HvTIP1.2* gene (HORVU3HR1G116790) encoding tonoplast intrinsic protein. The drought stress led to a decrease in the expression level of this gene for − 7.5 (log_2_FC) in ‘Sebastian’, − 7.9 (log_2_FC) in *hvsnac1.d* and − 6.5 (log_2_FC) in *hvsnac1.e* (Supplementary Materials, STable 2,3). Another example is the dehydrin 4 gene (HORVU6HR1G084070), which encodes a protein from a late embryogenesis abundant II genes group. The drought stress led to an increase in the expression level of this gene for 12.3 (log_2_FC) in ‘Sebastian’, 9.5 (log_2_FC) in *hvsnac1.d* and 11.8 (log_2_FC) in *hvsnac1.e*. This indicates severe drought stress under the tested conditions.

To take advantage of the analysis of both mutants, we focused on those drought-responsive DEGs groups that were specific to each mutant in comparison to WT. These two groups were compared together (Fig. 8D) and divided into three additional groups. The common group consisted of 90 genes (71 upregulated and 19 downregulated). Then, we concentrated on downregulated DEGs, as we expected that mutation in the *HvSNAC1* gene would interfere with the function of the HvSNAC1 protein, resulting in disturbances in the expression of its downstream target genes. This group was used to identify homologues in Arabidopsis, as the most extensively annotated genome, and analysed in terms of their predicted expression in guard cells. Next, we analysed the promoter sequences of chosen DEGs in terms of the presence of the CACG motif. Among these, possible targets for HvSNAC1 regulation were identified, which led to a better understanding of the action of HvSNAC1 in barley plants under drought stress.

### Genes downregulated by drought stress dependently on *hvsnac1* mutations

Among 19 downregulated DEGs between drought and control conditions, which were common for both mutants and specific only for them not for parent cultivar ‘Sebastian’ (Fig. 8D), five genes were excluded from the analysis (Table 3). One gene encoding auxin response factor (HORVU2Hr1G121110) was excluded because of the opposite change in expression level between mutants after drought stress compared with control conditions. The accession numbers of four genes (HORVU1Hr1G086160, HORVU1Hr1G035730, HORVU0Hr1G002100 and HORVU5Hr1G001580) did not match any record in the database (https://nov2020-plants.ensembl.org/Hordeum_vulgare/Info/Index). For eight genes, the homolog in the Arabidopsis genome has been identified (Ensemble database); for them, information on whether expression occurred in guard cells is known (TAIR database). The lack of expression in guard cells was detected only for one Arabidopsis homolog of the barley gene encoding profilin (HORVU1Hr1G075570) (Table 5). To identify HvSNAC1-responsive *cis*-elements in the promoter regions of the selected DEGs, 2 kb of the upstream sequences relative to the transcription start sites were searched according to the presence of CACG sequences. The promoters of a total of ten genes (HORVU4Hr1G046610, HORVU7Hr1G114400, HORVU6Hr1G033190, HORVU5Hr1G103990, HORVU3Hr1G075210, HORVU0Hr1G017960, HORVU7Hr1G098260, HORVU5Hr1G098920, HORVU3Hr1G068650 and HORVU6Hr1G012270) possess CACG motives in larger number of copies, which varied from three to 12 (Table 3). This suggests that HvSNAC1 may be involved in regulating their expression. In case of the promoters of four genes (HORVU1Hr1G075570, HORVU5Hr1G107240, HORVU2Hr1G118700 and HORVU2Hr1G013500), only one copy of CACG sequence was detected. Previous in vitro studies have shown that at least two CACG-motifs are necessary for SNAC1 binding, because it acts as dimer. Among the analysed genes, drought stress led to the highest decrease in the expression level of *HvEXPA8* (*expansin 8*, HORVU3Hr1G075210) for − 7.6 (log_2_FC) in *hvsnac1.d* and − 5.6 (log_2_FC) in *hvsnac1.e*.

**Fig. 9:**
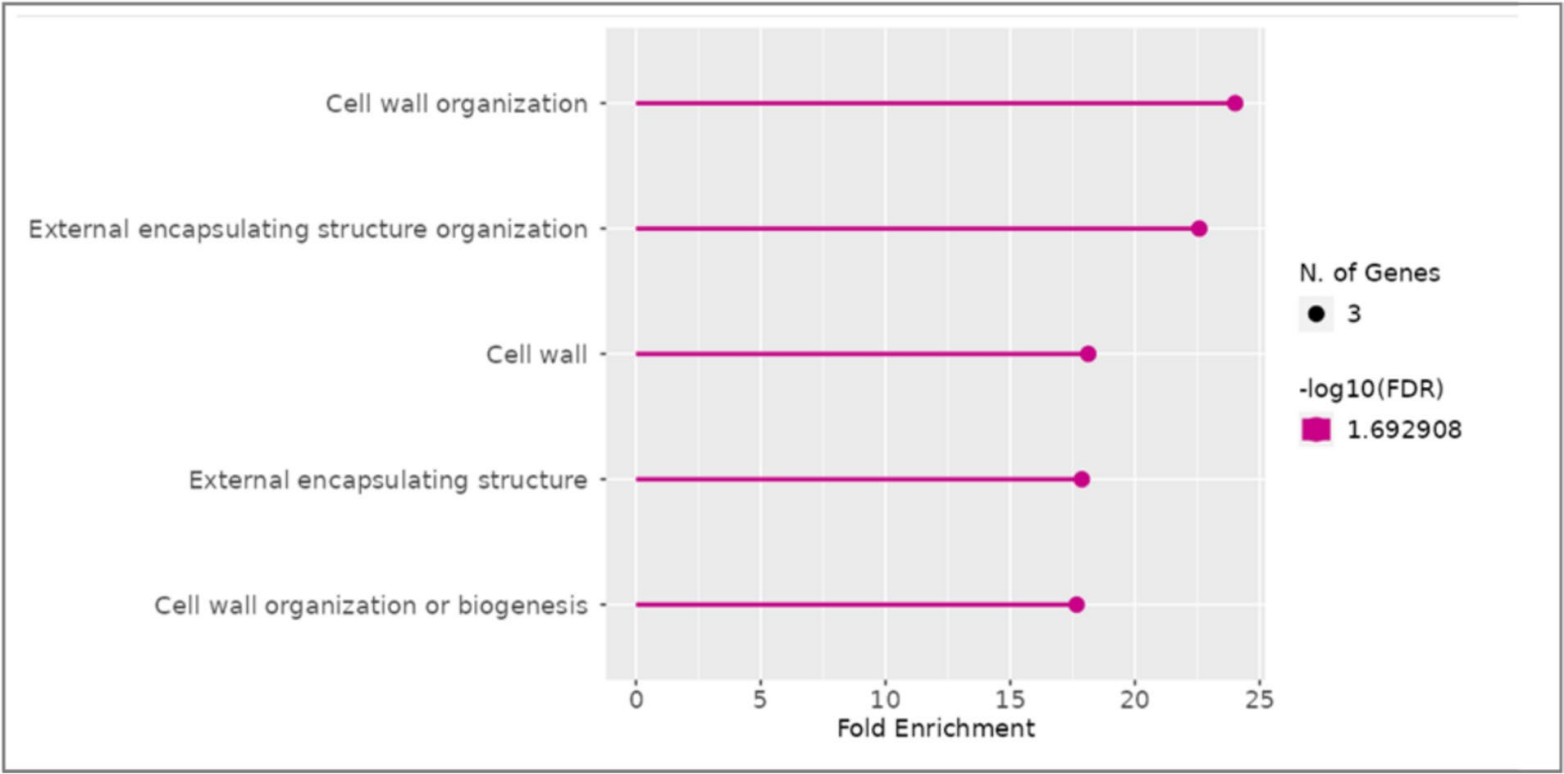
GO (gene ontology) enrichment analysis of common, downregulated differentially expressed genes (DEGs) for *hvsnac1.d* and *hvsnac1.e* between drought stress and control conditions

The specific, predicted roles of downregulated drought-responsive genes specific for mutants*,* but not for the parent cultivar were identified using a gene ontology (GO) enrichment study of the 19 DEGs. Based on their putative roles, they were divided into five pathways: cell wall organization, external encapsulating structure organization, cell wall, external encapsulating structure, cell wall organization and biogenesis (Fig. 9). The enrichment analysis led to the identification of the cell wall organization category and genes, such as *HvEXPA8 (expansin 8*), *HvXTH* (*xyloglucan endotransglucosylase/hydrolase*) or *HvPAE9* (*pectin acetylesterase 9*), as potential targets for HvSNAC1 regulation. This indicates that HvSNAC1 may be involved in regulating of genes associated with stomatal density and size.

Additionally, we conducted an analysis to identify transcription factors from the NAC family among the DEGs between control conditions and drought stress. We did not find any genes specific to both *hvsnac1.d* and *hvsnac1.e.* However, DEGs that showed either increased or decreased expression levels in the analysed genotypes (mutants separately, parent cultivar ‘Sebastian’ or common for each mutant and ‘Sebastian’) were detected. For some of these genes, potential orthologs in the Arabidopsis genome were identified, including genes with increased expression, such as *NAC002* and *NAC025*, and genes with decreased expression, such as *NAC030* and *NAC047* (Supplementary Materials, STable 4).

Another intriguing aspect of our study is the analysis of mutant-specific genes with upregulated expression. Investigating this group could provide valuable insights into a potential negative regulatory loop involving HvSNAC1. In our analysis, we identified 71 such genes (Fig. 8D; Supplementary Materials, STable 5). To further explore this group, we performed a GO enrichment analysis using the ShinyGO tool. This analysis revealed two potential pathways: sulfotransferase activity and transferase activity involving sulfur-containing groups (Supplementary Materials, SFigure 10). For genes associated with these pathways, potential orthologs in Arabidopsis genome were detected: *AtSOT17* (*sulfotransferase 17*) and *AtSOT16* (*sulfotransferase 16*) (Supplementary Materials, STable 6).

## Discussion

Since its first application in 2000, TILLING has remained an essential method for generating genetic diversity in genes of interest and plays a vital role in plant research, both for the functional analysis of genes and in breeding programs (Szurman-Zubrzycka et al. 2023). Here, we utilized TILLING to identify and phenotypically characterize new alleles of the *HvSNAC1* (*stress-responsive NAC 1*) gene emphasizing on its possible role in drought stress response. We used the *Hor*TILLUS Hordeum—TILLING—University of **S**ilesia (Hordeum) population of spring barley cultivar ‘Sebastian’ (Szurman-Zubrzycka et al. 2018), as a material for screening for the mutations. The *SNAC1* gene is one of many possible candidate genes for increased crop drought tolerance, suggested from studies that used transgenic plants with *SNAC1* overexpression in many plant species like rice, wheat, barley, cotton, maize, banana or oat led to better response under stress conditions (Cattivelli et al. 2008; Kurowska and Daszkowska-Golec 2023).

In this study, we identified 19 mutations in the analysed gene. The overall density of mutations was high (one mutation per 405 kb), what was slightly higher than the average mutation density in the *Hor*TILLUS population estimated from TILLING analysis of 32 different genes, 1/477 kb (Szurman-Zubrzycka et al. 2018). The latest research in other barley TILLING populations showed an even higher mutation density of 1/154 kb or 1/256 kb, but with the use of whole exome capture sequencing and whole genome sequencing, respectively (Schreiber et al. 2019; Jiang et al. 2022).

We chose two mutants with a missense mutation in the *HvSNAC1* gene and the parent cultivar ‘Sebastian’ to analyse their responses to drought stress, followed by a global transcriptome analysis. The 3D structures of the DNA-binding NAC domain consist of a central semi-*β*-barrel formed by seven twisted anti-parallel *β*-strands with three *α*-helices on one side and the open side (Chen et al. 2011). The analysed substitutions may impact the binding properties of HvSNAC1 to DNA because previous research has shown that the highly conserved positively charged subdomains C and D are crucial for binding to DNA (Duval et al. 2002; Olsen et al. 2005; Chen et al. 2011). The substitution in *hvsnac1.e* is placed very near the WKAT sequence (Fig. 1). A study conducted in ANAC19 showed an interaction with DNA via the *β*-sheet, which encompasses the conserved WKAT sequence (Welner et al. 2012). NACs bind DNA as dimers (Olsen et al. 2005), which modulates DNA-binding specificity (Müller 2001). Furthermore, it has been reported that NAC dimer flexibility is a possible mechanism for adapting to different spacings between binding sites, which may play a role in biological functions (Welner et al. 2012).

Our experiment comprised two primary phases: a control phase and a drought stress phase. During the control phase, plants were cultivated for 10 days at a temperature of 20 °C under optimal watering conditions, with soil moisture maintained at 12% volumetric water content (vwc). The drought stress phase spanned a total of 14 days. In the initial 4 days of this phase, soil moisture was reduced to 3% vwc while maintaining a temperature of 20 °C. For the subsequent 10 days, plants were grown at 25 °C, with soil moisture further reduced to a range of 3–1.3% vwc. This experimental design accounted for varying temperature conditions to mimic the environmental variability that plants experience in nature. Drought stress in natural environments is often associated with rising temperatures, and our study aimed to simulate this interaction. To cope with drought, plants may reduce stomatal density or stomatal size, conserving water in the process. Conversely, during heat stress, plants may defend themselves by increasing transpiration through enhanced stomatal density, which helps to cool their leaves. However, plant species could have different stomatal developmental responses to drought and high temperatures (Daszkowska-Golec and Szarejko 2013; Matkowski and Daszkowska-Golec 2023; Chua and Lau 2024). We detected two main differences between the mutants and WT under phenotyping. First, the density and morphology of stomata differed both under control conditions and after drought stress (Fig. 4). Second, we detected changes in ABA sensitivity of mutants to stomata movements: faster and long-lasting closure of stomata after ABA spraying of leaves (Fig. 5). The mechanism of correct stomatal movement is impaired in mutants. After stress, plants need to reopen their stomata as soon as possible and return to growth by starting transpiration. HvSNAC1 might be engaged not only in stomatal closure but also in stomatal reopening. Hu et al. (2006) showed that rice transgenic lines overexpressing *SNAC1* were more ABA-sensitive during germination and seedling growth (Li et al. 2019). ChIP-Seq analysis reveals that SNAC1 binds to CACGT and CACGTA under normal growth conditions and to ACGTGG under drought conditions, all of which possess the core motif of the ABA-responsive element (ABRE)-ACGT (Li et al. 2019). SNAC1 can bind in vitro to the promoter sequence of *OsbZIP23*, an important ABA signaling regulator, and regulates *OsNCED3* (*9-cis-epoxycarotenoid dioxygenase*), a key enzyme in the biosynthesis of ABA (Li et al. 2019). All these results indicate that SNAC1 acts in an ABA-dependent manner and may be involved in signaling cascades by which plants promote stomatal closure by activating ABA biosynthesis. Till now, no research has indicated that SNAC1 is involved in the control of stomatal density and morphology. Both mutants exhibited higher stomatal density after drought stress than the parent cultivar ‘Sebastian’, whereas only *hvsnac1.d* presented higher stomatal length unlike to *hvsnac1.e* (Fig. 4). The global transcriptome analysis of *hvsnac1.d* and *hvsnac1.e* mutants was directed toward the identification of candidate genes that may be regulated by HvSNAC1 under drought stress conditions. Thus, we focused our analysis on drought-related DEGs that were specific only for both mutants. Because we assumed that HvSNAC1 did not act correctly in these mutants, we investigated downregulated DEGs to identify putative target genes of HvSNAC1. Among these DEGs, we identified genes connected with cell wall organization, such as *HvEXPA8* (*expansin 8*), *HvXTH* (*xyloglucan endotransglucosylase/hydrolase*) or *HvPAE9* (*pectin acetylesterase 9*). Expansin is a non-enzymatic cell wall-loosening protein that causes damage to hydrogen bonds between xyloglucan and cellulose microfibrils (Lu et al. 2013). The overexpression of a rose expansin gene- *RhEXPA4* in Arabidopsis led to decreased stomatal density under drought stress (Lü et al. 2013). In our study, downregulation of *HvEXPA8* was correlated with opposite phenotype: increased stomatal density in both mutants after drought stress compared with the parent cultivar ‘Sebastian’. Wall loosening is an essential step in guard cell swelling. In Arabidopsis, lines overexpressing *AtEXPA1* present an increase in the rate of light-induced stomatal opening (Wei et al. 2011; Zhang et al. 2011). Here, we report that mutants with downregulated *HvEXPA8* showed impaired stomatal opening. Additionally, high levels of expansin proteins may acidify the cell wall and promote the growth rate of the stem, as observed in *Rumex palustris* (Vreeburg et al. 2005). The xyloglucan endotransglucosylase/hydrolase (XTH) family responds to cleaving and reconnection of xyloglucan molecules. These enzymes play a role in cell wall loosening for plant cell expansion (Ishida and Yokoyama 2022). To the author’s knowledge, no study shows the involvement of this enzyme in regulating stomatal movement. Still, they might play similar roles as expansin, as both lead to cell wall loosening. An increased stomatal density observed in the investigated mutants could be associated with the downregulation of the identified *HvXTH* as well as impaired stomatal reopening. Pectin acetyl esterases (PAEs) affect pectin acetylation by hydrolyzing acetyl ester bonds and the amount and distribution of O-acetylation (Shahin et al. 2023). No study has shown the direct effect of changes in PAE activity on the stomatal phenotype. However, there is some indirect evidence, e.g., Gou et al. (2012) reported the association of decreased acetylation in *Nicotiana tabacum* with wilting, and Stranne et al. (2018) with elevated levels of drought tolerance in Arabidopsis. The observed phenotype may be related to the stomatal action.

Among the downregulated DEGs specific for mutants, we identified a gene encoding a protein from the kinase superfamily. Blue light causes stomatal opening in most plants through a signaling network that involves protein kinases, which phosphorylate and activate H^+^-ATPase in the plasma membrane of guard cells (Kinoshita et al. 1999). In our study, mutants with downregulation of genes encoding proteins from the kinase superfamily presented impairment in stomatal opening.

We also detected a lower nitrogen balance index (NBI) under drought in *hvsnac1.d* mutant comparing to WT, which is a good indicator of plant nitrogen status (Figure S2). Recently, Qi et al. (2023) showed that SNAC1 regulates the expression of *OsNRT2.1* (*nitrate transporter 2.1*) and promotes nitrate uptake. *SNAC1*-overexpression resulted in higher N uptake, NUE (nitrogen use efficiency) and N use index (NUI), whereas mutation in *SNAC1* generated by CRISPR/Cas9 technology resulted in decreased N uptake and lower NUI (Qi et al. 2023).

## Conclusions

The present results indicated that barley *hvsnac1.d* and *hvsnac1.e* mutants exhibit changes in the density and size of stomata under drought and are characterized by impaired stomatal reopening after exogenous treatment with ABA. RNA-seq transcriptome analysis provides new data regarding the function of HvSNAC1 in the drought response in barley. Specifically, we found downregulation of genes associated with cell wall organization, such as *HvEXPA8* (*expansin 8*), *HvXTH* (*xyloglucan endotransglucosylase/hydrolase*) or *HvPAE9* (*pectin acetylesterase 9*) existed. Additionally, *hvsnac1.d* showed reduced nitrogen status, as indicated by a lower nitrogen balance index (NBI) than WT under drought stress. Together, these findings increase our understanding of SNAC1-dependent modulation of plant responses to abiotic stress. Further analysis is required to confirm the interaction between HvSNAC1 and the promoters of its putative target genes.

## Supplementary Information

Below is the link to the electronic supplementary material.

Supplementary file1 (DOCX 4925 KB)

Supplementary file2 (XLSX 1147 KB)

Supplementary file3 (XLSX 424 KB)

## Data Availability

The datasets generated and analyzed during the current study are available from the corresponding author upon reasonable request.

## Abbreviations

ABA: Abscisic acid
AbA: Aureobasidin A
ABS/CS: Energy absorbed per excited CS
ADP: ADP-ribozylation factor 1
AUX: Auxin
AWD: Adaptation to water deficit
CG: Control growth
ChIF: Chlorophyll *a* fluorescence
ChIP-Seq: Chromatin immunoprecipitation sequencing
CS: Cross-section
DEGs: Differentially expressed genes
DI_0_/CS: Energy of dissipation per CS
DRE: Dehydration-responsive element
DS: Drought stress
ERD1: Early responsive to drought 1
ET_0_/CS: Electron transport per CS
EXPA8: Expansin 8
*F*_*v*_/*F*_*m*_: Maximum quantum yield of photosystem II
GA: Gibberellins
HM: Homozygous state
HT: Heterozygous state
*Hor*TILLUS: *Hordeum vulgare* – TILLING – University of Silesia
LTR: Low temperature-responsive elements
MBS: MYB binding site
MeJA: Methyl jasmonate
MNU: N-methyl-N-nitrosourea
NACRS: NAC recognized sequence
NAM: No apical meristem
NaN_3_: Sodium azide
NBI: Nitrogen balanced index
NRT2.1: Nitrate transporter 2.1
NUE: Nitrogen use efficiency
NUI: Nitrogen use index
ORF: Open reading frame
PAE9: Pectin acetylesterase 9
PARSESNP: Project aligned related sequences and evaluate SNPs
PI_ABS_: Performance index per absorption
PlantCARE: Plant cis-acting regulatory elements
PP18: Protein phosphatase 18
PSI: Photosystem I
PSII: Photosystem II
RC: Reaction center
RC/CS: Density of active reaction centers per CS
RE_0_/CS: Electron flux reducing end electron acceptors at the PSI acceptor side
RNA-seq: RNA sequencing
RWC: Relative water content
SIFT: Sorting intolerant from tolerant
SNAC: Stress-responsive NAC1
SEM: Scanning electron microscope
SRO1c: Similar to RCD 1
TAR: Transcriptional activation region
TDR: Time-domain reflectometer
TFs: Transcription factors
TILLING: Targeting induced local lesion IN genome
TR_0_/CS: Energy trapped per CS
WT: Wild-type
XTH: Xyloglucan endotransglucosylase/hydrolase
Y1H: One-hybrid assay
