# Supplementary material for: Functional analysis of HvSNAC1 in stomatal dynamics and drought adaptation
Source: J Appl Genet. 2025 Mar 18;66(4):817–40. doi: 10.1007/s13353-025-00956-6 (PMC12605542; doi:10.1007/s13353-025-00956-6)
Supplement: Supplementary file 1 — Supplementary file1 (DOCX 4925 KB) [file 13353_2025_956_MOESM1_ESM.docx]

Supplementary Materials


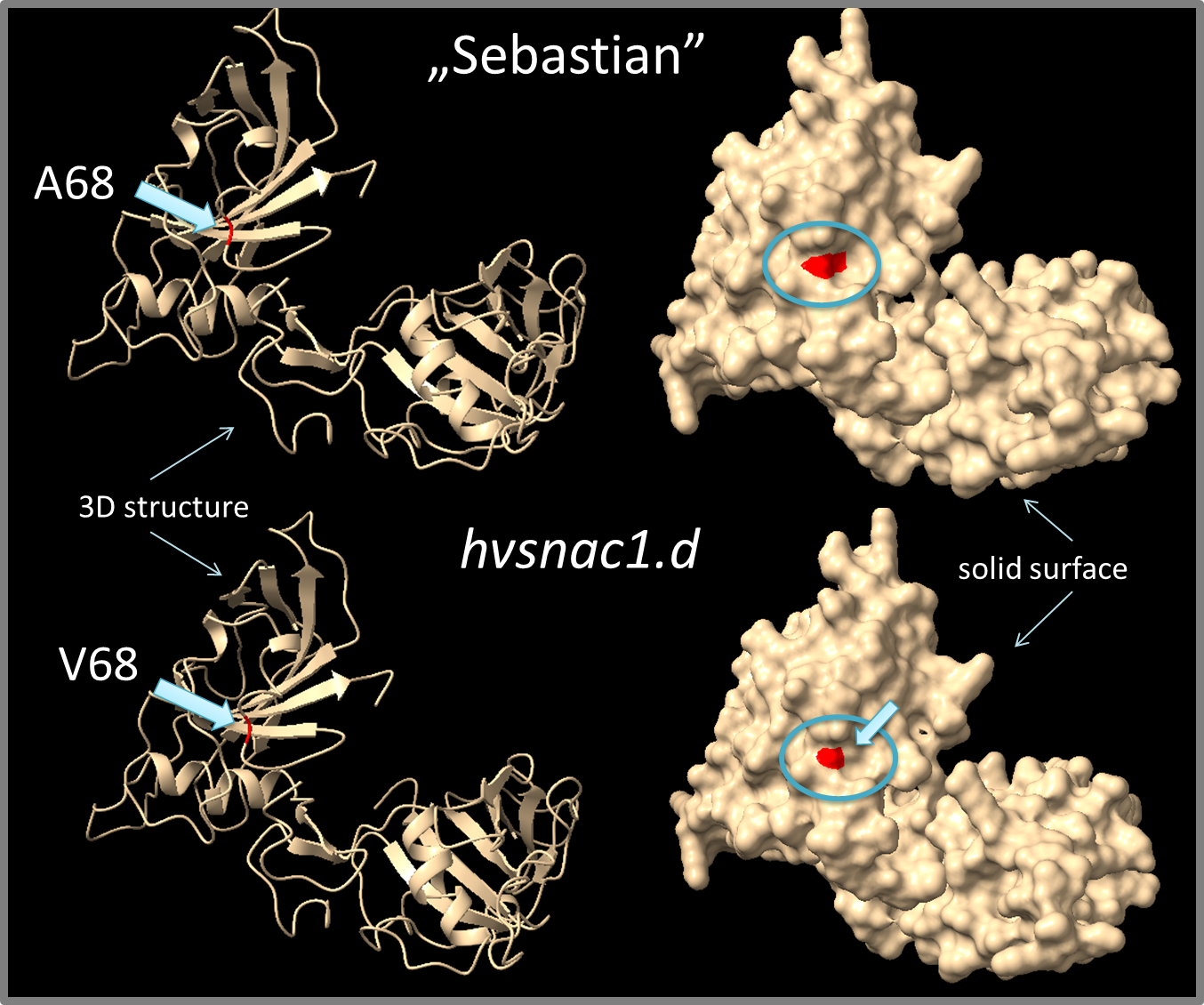


**SFigure 1.** The 3D structure prediction of the HvSNAC1 protein from the parent cultivar ‘Sebastian’ and the mutant *hvsnac1.d* was performed using ChimeraX 1.9. The mutant carries a substitution at position 68 in the amino acid sequence, where alanine is replaced by valine (A68V). This substitution causes slight alterations in the protein's surface topology, which are highlighted by a blue circle.


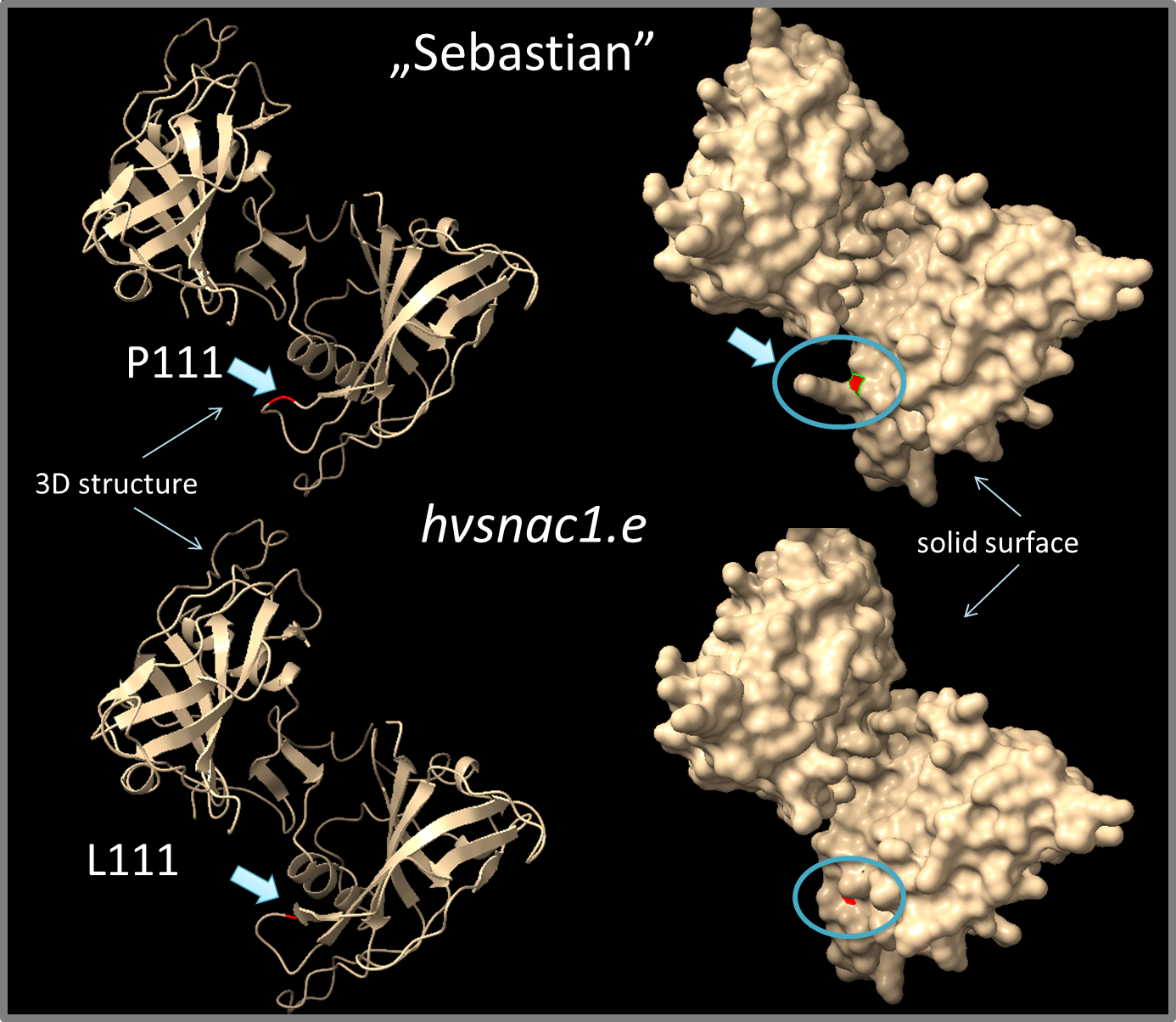


**SFigure 2.** The 3D structure prediction of the HvSNAC1 protein from the parent cultivar ‘Sebastian’ and the mutant *hvsnac1e* was performed using ChimeraX 1.9. The mutant carries a substitution at position 111 in the amino acid sequence, where proline is replaced by leucine (P111L). This substitution causes significant alterations in the protein's surface topology, which are highlighted by a blue circle.


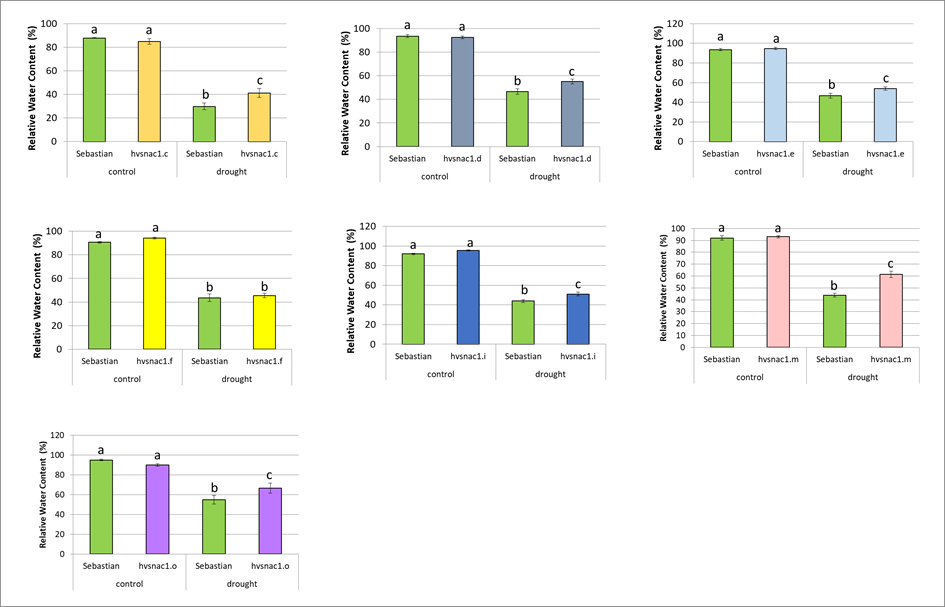


**SFigure 3.** Relative Water Content (RWC in %) of parent cultivar ‘Sebastian’ and mutants: *hvsnac1c,* *hvsnac1d,* *hvsnac1e* *hvsnac1f,* *hvsnac1i,* *hvsnac1m* and *hvsnac1o* in control condition, and after 10 days of drought treatment assessed in different experiments Tested forms carrying mutation in homozygous stage The values are presented as the means ± SE of nine plants per one biological replication, and three biological replications were used Statistically significant differences between different genotypes and growth conditions were assessed using a one-way ANOVA followed by the Fisher Least Significant Difference (LSD) test (p < 005) and are indicated by different letters


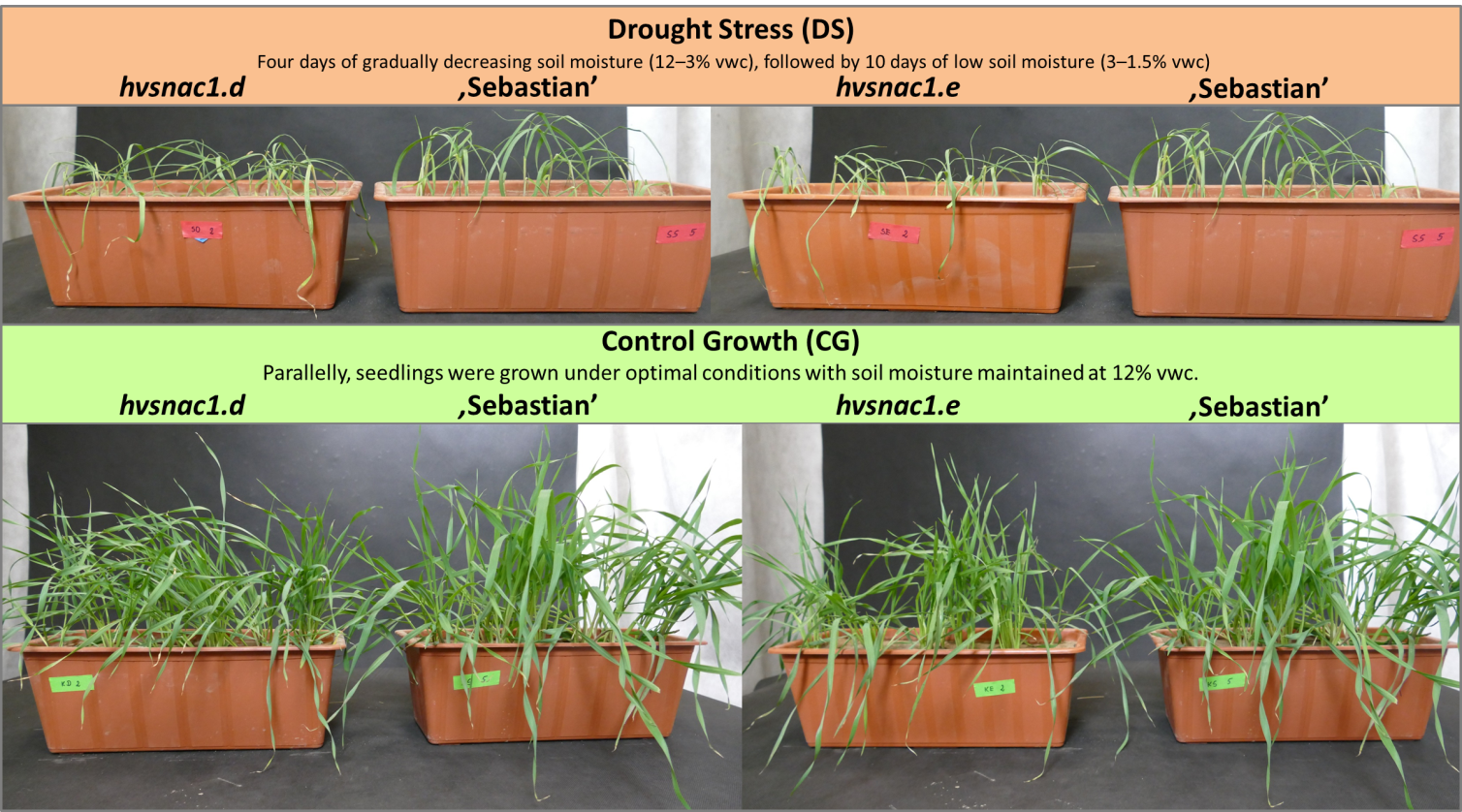


**SFigure 4.** Appearance of the parent cultivar ‘Sebastian’ and mutants (*hvsnac1.d, hvsnac1.e*) under control conditions and after 10 days of drought treatment.


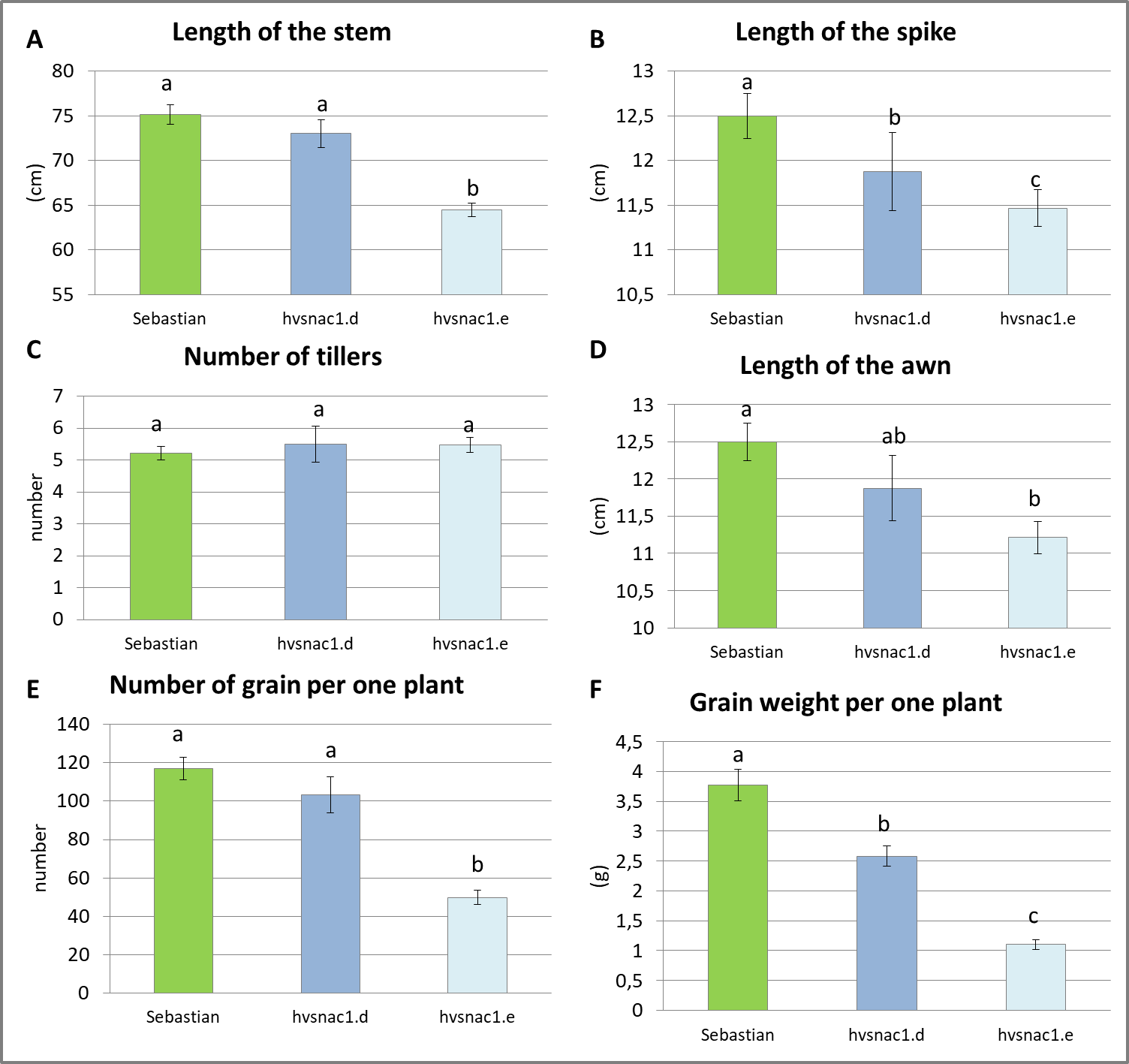


**SFigure 5.** Post-harvest analysis of plant growth under controlled conditions for ‘Sebastian’, *hvsnac1.d* and *hvsnac1.e,* evaluating the following parameters: stem length (A), spike length (B), number of tillers (C), awn length (D), number of grains per plant (E), and grain weight per plant (F). Statistically significant differences between genotypes were assessed using one-way ANOVA, followed by Fisher’s Least Significant Difference (LSD) test (p < 0.05), with different letters indicating significant differences.


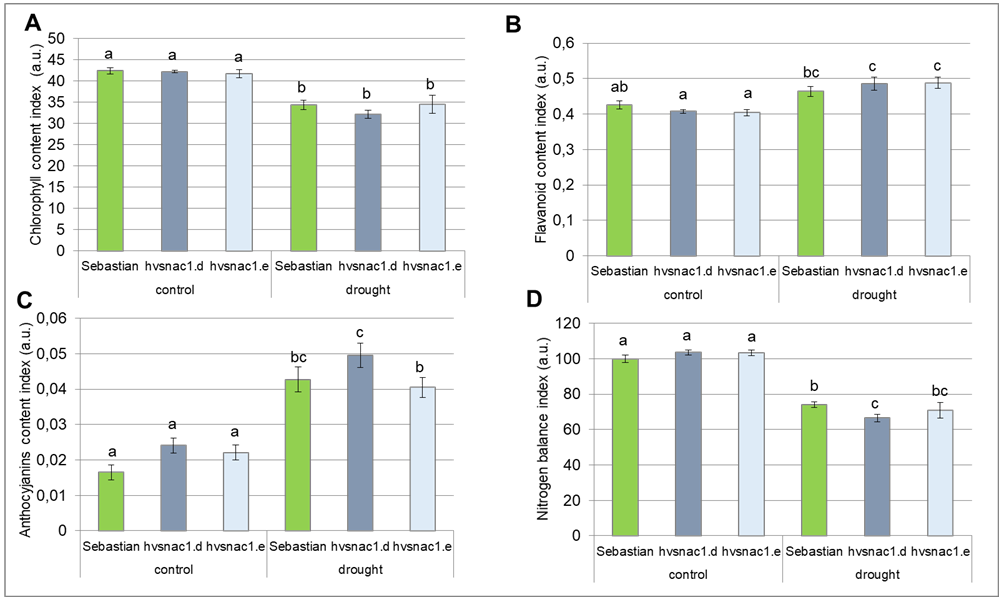


**SFigure 6.** Chlorophyll (A), flavonoid (B), anthocyanins (C) content indexes, and Nitrogen Balance Index (NBI) of parent cultivar ‘Sebastian’ and mutants (*hvsnac1d, hvsnac1e*) in control condition, and after 10 days of drought treatment The values are presented as the means ± SE, nine plants per one biological replication, three biological replications were used Statistically significant differences between different genotypes and growth conditions were assessed using a one-way ANOVA followed by the Fisher Least Significant Difference (LSD) test (p < 005) and are indicated by different letters


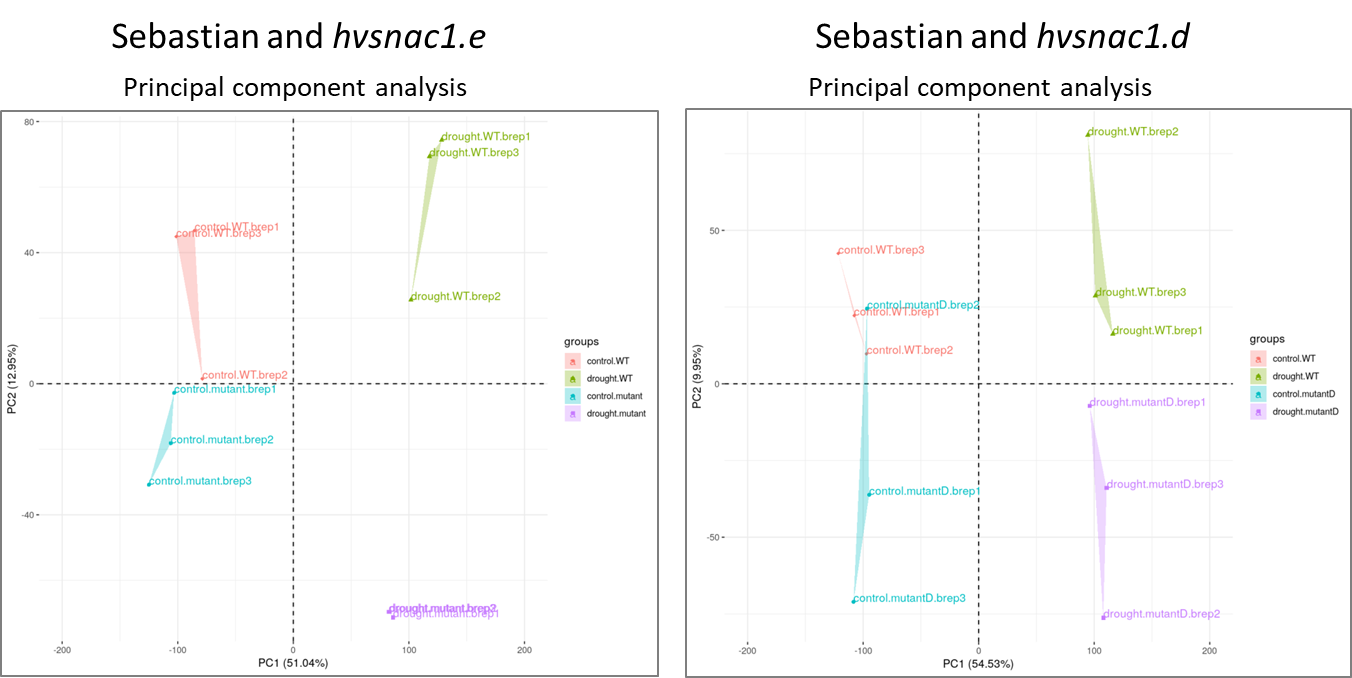


**SFigure 7.** Principal component analysis (PCA) for transcriptomes of barley leaves of parent cultivar ‘Sebastian’ and mutants (*hvsnac1.e, hvsnac1.d*) in the control condition and after ten days of drought (one dot symbolizes one repetition).


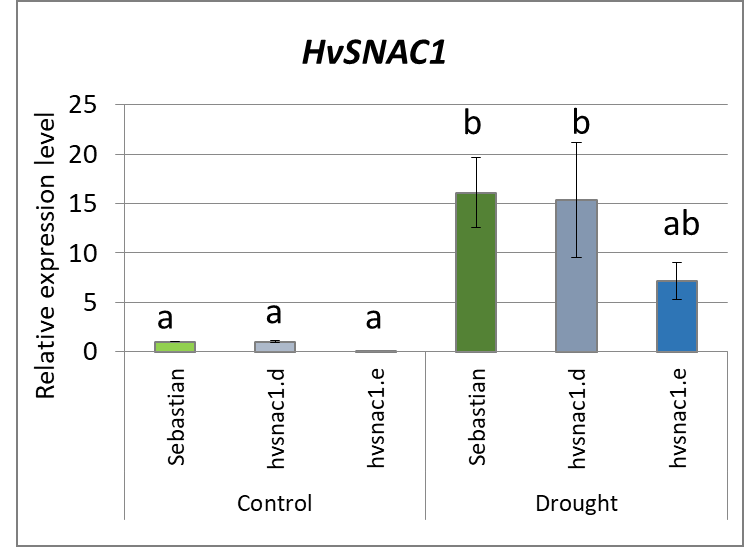


**SFigure 8.** Expression patterns of *HvSNAC1* in parent cultivar ‘Sebastian’ and mutants in control condition and after ten days of drought. Relative expression level was evaluated in comparison to WT which were grown under optimal soil moisture (control) Statistically significant differences between different genotypes and growth conditions were assessed using a one-way ANOVA followed by the Fisher Least Significant Difference (LSD) test (p < 005) and are indicated by different letters.


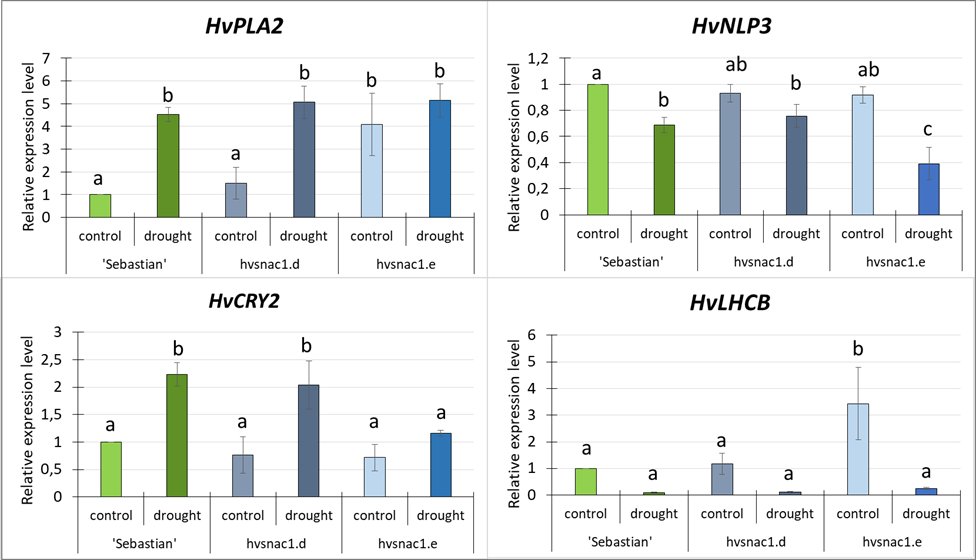


**SFigure 9** Expression patterns of four randomly selected genes in parent cultivar ‘Sebastian’ (WT) and mutants in control condition and after ten days of drought *HvPLA2 (Phospholipase A2-alpha)*, HORVU4Hr1G061740; *HvNLP2* (*Plant regulator RWP-RK family protein),* HORVU3Hr1G032170; *HvCRY2* (*Cryptochrome-2),* HORVU6Hr1G058730; *HvLHCB (Chlorophyll a-b binding protein)*, HORVU6HR1G091650. Relative expression level was evaluated in comparison to WT which were grown under optimal soil moisture (control). Statistically significant differences between different genotypes and growth conditions were assessed using a one-way ANOVA followed by the Fisher Least Significant Difference (LSD) test (p < 005) and are indicated by different letters


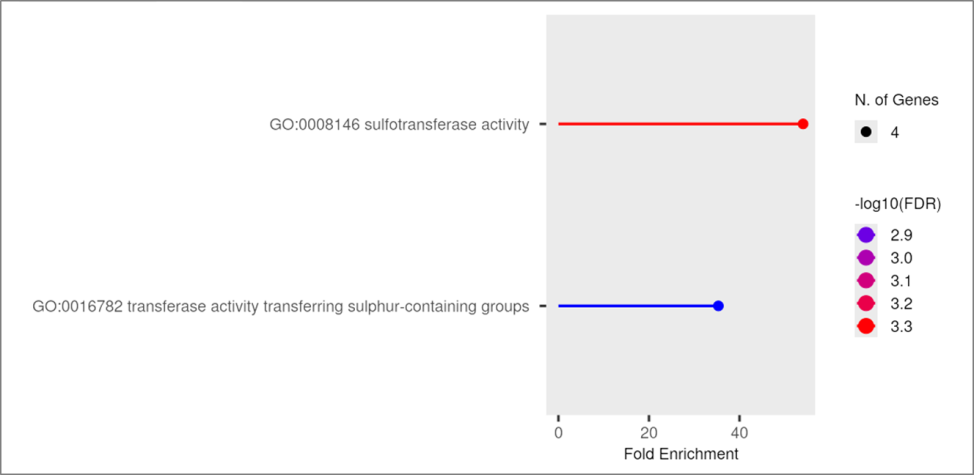


SFigure 10. GO (Gene Ontology) enrichment analysis of common, upregulated differentially expressed genes (DEGs) for *hvsnac1.d* and *hvsnac1.e* between drought stress and control conditions.

**STable 1** Selected parameters related to photosynthesis efficiency measured in leaves of parent cultivar ‘Sebastian’ and two mutants (*hvsnac1d, hvsnac1e*) and in control condition, and after 10 days of drought treatment Means ± SE are presented for each of the analysed parameters Statistically significant differences (p < 005) are indicated by different letters (a–e) Differences only within each condition between WT and mutant are indicated in green, while between mutants in violet

| **Conditions** | **Genotype** | **ABS/CS_0_** | **DI_0_/CS_0_** | **TR_0_/CS_0_** | **ET_0_/CS_0_** | **RE_0_/CS_0_** | **F_v_/F_m_** | **RC/CS_0_** | **PI _ABS_** |
| --- | --- | --- | --- | --- | --- | --- | --- | --- | --- |
| control | Sebastian | 5424±91ab | 923±22a | 4501±70ab | 2827±64a | 1332±25a | 0,83±0,002a | 2890±115ab | 4,3±0,2a |
|  | *hvsnac1d* | 5279±106a | 922±18a | 4357±91a | 2817±44a | 1262±71ab | 0,83±0,002a | 2808±62abc | 4,7±0,2a |
|  | *hvsnac1e* | 5434±68ab | 930±19a | 4504±51ab | 2811±42a | 1168±43bc | 0,83±0,002a | 2943±93a | 4,4±0,2a |
| drought | Sebastian | 5673±116bc | 1138±37b | 4535±81ab | 2622±80b | 931±27e | 0,80±0,003bc | 2559±78cd | 2,5±0,2bc |
|  | *hvsnac1d* | 5697±70c | 1180±21b | 4517±60ab | 2613±61b | 1001±40de | 0,79±0,003b | 2489±83d | 2,4±0,2b |
|  | *hvsnac1e* | 5755±69c | 1142±28b | 4613±54b | 2817±75a | 1114±45cd | 0,80±0,004c | 2639±100bcd | 3,0±0,3c |

ABS/CS, absorption energy flux per cross-section (CS) of leaf area; DI_0_/CS_0_, dissipation energy flux per CS; TR_0_/CS_0_, trapped energy flux per CS; ET_0_/CS_0_, electron transport flux per CS; RE_0/_CS_0_, electron flux reducing end electron acceptors at the photosystem I (PSI) acceptor side; F_v_/F_m_, maximum quantum yield of photosystem II (PSII); RC/CS, number of active reaction centers per illuminated CS; PI_ABS_, performance index on absorption basis, based on (Strasser et al, 2004)

**STable 2.** DEGs between control and drought stress in ‘Sebastian’ and *hvsnac1.d*

**STable 3.** DEGs between control and drought stress in ‘Sebastian’ and *hvsnac1.e*

**STable 4.** NAC transcription factors, which have been identified as DEGs between control and drought stress in different genotypes

*high confidence; in orange, up-regulated and in blue, down-regulated DEGs have been marked

**STable 5.** Drought-responsive DEGs, upregulated and specific only for mutants *hvsnac1.d* and *hvsnac1.e* under 10 days drought stress

**STable 6.** The selected drought-responsive DEGs, which are upregulated and specific to the *hvsnac1.d* and *hvsnac1.e* mutants under 10 days of drought stress, belong to categories identified through GO enrichment analysis: sulfotransferase activity and transferase activity involving sulfur-containing groups
